# Adhesive Protein Nanoparticles Control Emulsion Properties via Tunable Interdroplet Linking

**DOI:** 10.1002/smll.75117

**Published:** 2026-08-12

**Authors:** Gijs D. Konings, Ketan A. Ganar, Emmanouil Chatzigiannakis, Elke Scholten, Constantinos V. Nikiforidis

**Affiliations:** ^1^ Biobased Chemistry and Technology Wageningen University Wageningen Netherlands; ^2^ Physics and Physical Chemistry of Foods Wageningen University Wageningen Netherlands; ^3^ Processing and Performance Group, Mechanical Engineering Department Eindhoven University of Technology Eindhoven Netherlands; ^4^ Institute of Complex Molecular Systems Eindhoven University of Technology Eindhoven Netherlands

**Keywords:** 3D printing, adhesive nanoparticles, interfacial rheology, microfluidics, thin film dynamics, tunable droplet linking

## Abstract

The mechanical properties of emulsions are governed by the ability of droplets to resist shear, making interdroplet connections crucial for tuning emulsion behavior. Here, we elucidate the mechanism by which dispersed oil droplets can be “glued” together using pea‐protein nanoparticles that self‐assemble through electrostatic interactions. We experimentally demonstrate the adhesive action of these nanoparticles across multiple length scales, using microfluidics, the thin film balance, confocal microscopy, and rheology, and explain the physical mechanism using theoretical models. Our results show that the nanoparticles simultaneously bind to two opposing droplet interfaces, forming electrostatically driven bridges that link droplets together. This adhesion dramatically transforms the mechanical response of the emulsion from a liquid‐like material to a plastic‐like, 3D‐printable system, raising the storage modulus of emulsions from < 0.1 to 3.2 kPa. These insights highlight the potential of biosourced nanoparticles as a powerful tool for engineering emulsion mechanics via electrostatic forces, enabling applications that require materials with controlled mechanical properties, such as fat tissue mimetics or soft microelectronics.

## Graphical abstract

In this study, pea protein nanoparticles were probed for their ability to link emulsion droplets. Experiments across multiple length scales showed that the nanoparticles form shear‐resistant links via bridging interactions from the thin film between droplets. The formation of interdroplet links can be steered via ionic strength, thus allowing tunable mechanical properties in emulsions, from liquid‐like to a 3D‐printable solid.

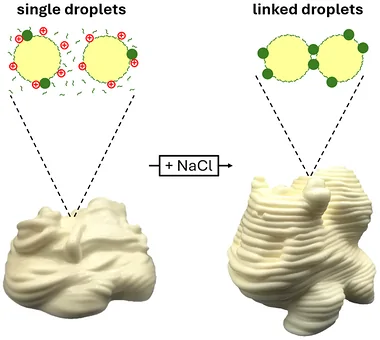

## 1 Introduction

Emulsions are inherently unstable systems prone to coalescence and phase separation, which originates from a thermodynamic drive to minimize the interfacial area between two immiscible fluids, like water and oil. Inclusion of surface active molecules, often amphiphilic [1, 2], grants a prolonged stability to the emulsions [2, 3]. Such surfactants adhere to the oil–water interfaces, reducing interfacial tension and often further promote stability by inhibiting coalescence through steric and/or electrostatic repulsion between droplets [4]. Through choosing the type of surfactants and the properties of the mixed fluids, emulsions with varying morphologies and mechanical properties may be created, such as o/w, w/o, and w/w emulsions [5], high internal phase emulsions [4, 6, 7, 8, 9], double emulsions [5, 10], and many more. These different types of emulsions find applications in a wide range of products, including cosmetics, pharmaceuticals, and food [1, 4, 11]. Each application has its own requirement with regard to the mechanical properties of the emulsion.

The product requirement might be related to a desired thickness, i.e., the gel strength, which can be achieved by reducing the mobility of emulsion droplets through an increased droplet volume fraction or increased viscosity of the continuous phase [4]. However, for some applications, like mimicking animal fat tissue, simple jamming of emulsion droplets does not offer enough mechanical strength, and further fortification is needed. Additional mechanical strength could be obtained by attractive interactions between droplets. At rest, attractive droplet interactions turn the emulsion into a solid‐like network of linked droplets, whereas high shear forces can limit such interactions, turning it into a liquid dispersion [12]. The strength of such links between emulsion droplets alter the microstructural arrangement of the droplets (aggregated vs. dispersed) and directly affect the rheological behavior [4, 13]. Reduced mobility due to droplet linking would promote elasticity with respect to gel strength, flow behavior, etc [14, 15].

In recent works from our team, it was shown that pea proteins can be used to provide sufficient attractive interactions to gain such shear‐responsive behavior [16]. However, this behavior was only obtained at pH 3, where the pea proteins self‐assembled into nanoparticles [16, 17, 18]. To reach pH 3, the proteins pass through the iso‐electric point (pH 4.8). At this point, the balance between electrostatic repulsion, and attractive hydrophobic and van der Waals forces, allows the proteins to self‐assemble [17]. Although large particles (≳1 µm) are also formed in the process, homogenization leads to deagglomeration of nanoparticle clusters, leaving nanoparticles with a distribution of 50 to 700 nm [17] or 50 to 500 nm [18]. When adsorbed on oil droplet interfaces, these nanoparticles have distinct adhesive properties [17, 19]. For jammed emulsions, where more droplet‐droplet contacts may occur, the nanoparticles appear to increase the mechanical strength of the emulsions (∼5 × higher $G^{\prime }$) [16]. The origin of this increase in mechanical strength is as of yet unknown. However, we suspect that additional mechanisms are at play other than simple droplet flocculation or jamming of the protein aggregates present in the continuous phase. The weak attractive forces responsible for droplet flocculation are easily overcome by shear. Even a jammed emulsion at high volume fraction ($\phi_{\mathrm{oil}}=$ 0.74) would have ample void space for the protein aggregates to reside in at the relevant protein concentration (1–2 wt%), thus preventing a significant jamming effect of the aggregates as passive fillers. Instead, if the nanoparticles were adsorbed simultaneously to two neighboring droplets, they would form a much stronger link between droplets ($\gg k_{B}T$), as discussed in literature [20, 21]. Understanding the nature of the driving force of droplet adhesion enables the application of a transition in rheological properties through an external stimulus. Using an external stimulus, droplet adhesion may be promoted and the emulsion's droplet interactions may be tuned to obtain the desired mechanical properties.

Pea protein nanoparticle‐mediated bridging of emulsion droplets is as of yet an unexplored subject in literature. Previous literature on the role of pea proteins in structuring emulsions has mainly focused on changes in the physicochemical properties of pea proteins with pH [22, 23, 24, 25] and how this affects emulsifying capacity. However, little to no attention has been paid to droplet‐droplet interactions as a result of physicochemical or conformational changes to the proteins imparted by pH. For the mixed monomeric protein‐particle system, the protein nanoparticles are hypothesized to aid in emulsion structuring by droplet‐droplet bridging interactions, while the monomeric proteins stabilize the o/w interface [17]. Such particle‐mediated droplet bridging is a well‐studied phenomenon for synthetic particles [26, 27, 28, 29], but these studies typically involve emulsion systems stabilized exclusively by Pickering particles. Whether particle‐mediated bridging occurs similarly for a mixed monomeric‐particle system of proteins will be explored in this study. Furthermore, utilization of a natural alternative like plant proteins would be favorable in terms of nutritional value and from a sustainability perspective. Pea proteins specifically are especially attractive because they are abundant, inexpensive, gluten‐free, and non‐allergenic [30, 31].

In this study, we used a multi‐scale approach, spanning interfacial rheology, thin film dynamics and bulk emulsion properties, to determine the mechanism and driving forces behind protein nanoparticle‐mediated droplet linking. By isolating repulsive versus attractive intermolecular forces, we could deduce the origin of the adhesive effect of nanoparticles on emulsion droplets and propose a global mechanism for interdroplet linking. The suggested mechanism was experimentally verified across different length scales and was also validated using theoretical models. With our findings, we present a non‐invasive strategy to tune emulsion mechanical properties from liquid‐like to solid‐like using ionic strength, and thereby allow for emulsions that can mimic the complex mechanical properties of animal fat tissue. Furthermore, the ion‐induced transition makes these emulsions suitable for 3D‐printing and opens the door to applications with intricate, tunable geometrical designs, such as soft microelectronics [32].

## 2 Results and Discussion

### 2.1 Protein Nanoparticle Formation and Their Effect on Bulk Rheology

To distinguish the effect of protein nanoparticles on the mechanical properties of an emulsion from that of individual proteins, two conditions were used: pH 3 and 7. Pea protein isolate dispersed at pH 3 or 7 yielded a roughly equivalent absolute surface potential of 27.6±2.1 or –25.4±2.7 mV, respectively. However, the protein dispersions were at different states of aggregation, with mainly individual proteins and only a few aggregates at pH 7 (too few scattered photons detected for an accurate size distribution measurement). Meanwhile, reducing the pH to 3 triggered the self‐assembly of proteins into nanoparticles with a Sauter mean diameter ($D_{3,2}$) of 150.6±8.8 nm (Figure 1a), similar to previously reported sizes [17, 18]. The formation of nanoparticles was likely induced by hydrophobic interactions, while electrostatic repulsion was reduced as the proteins in dispersion passed through the iso‐electric point during acidification. Adding oil to the protein dispersions at either pH and subsequent homogenization resulted in emulsions with a similar droplet size ($D_{3,2}\approx$ 2 µm). The emulsion morphology and droplet size distribution are given in Figure S1. Confocal microscopy of the emulsions revealed that at pH 7, the oil droplets were enveloped by a thin membrane of proteins and no aggregates were observed. In contrast, at pH 3, also distinct protein particles were present, trapped between the oil droplets (arrows, Figure 1b). Due to the resolution limit of the microscope (170 nm), not the complete range of protein particle sizes (30–800 nm) could be observed in Figure 1b, but only the protein particles with a size larger than the resolution limit. Although this cannot be directly observed in the confocal micrographs taken at rest, it is likely that the nanoparticles observed in the Plateau borders would become trapped in the films when shear was applied. Furthermore, our team previously reported that the protein nanoparticles reside at the interfaces of the oil droplets, together with individual proteins [17].

**FIGURE 1:**
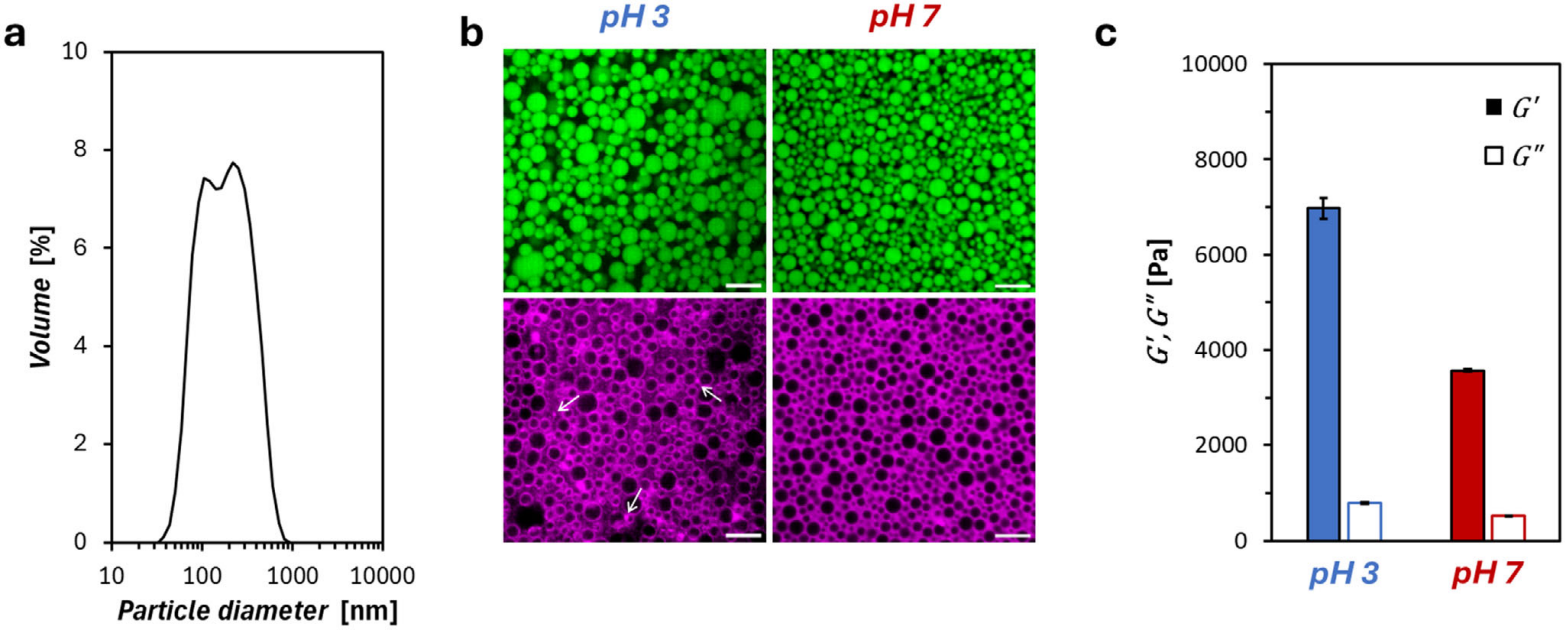
(a) Particle size distribution of nanoparticles self‐assembled at pH 3. (b) Confocal laser scanning micrographs showing the morphology of emulsions with (pH 3) and without (pH 7) nanoparticles (scale bar = 10 µm). (top) Lipid droplets stained with BODIPY 493/503. (bottom) Protein on or near the lipid droplet interface stained by Rhodamine B and arrows highlighting the location of nanoparticles. (c) Storage ($G^{\prime }$) and loss ($G^{\prime \prime }$) moduli in the linear viscoelastic regime (0.1% strain) for emulsions with (pH 3) and without (pH 7) nanoparticles. The emulsions were made with 2 wt% protein and had an oil volume fraction of 0.75.

The mechanical properties of the two emulsions (pH 3 and 7) were distinctly different despite their identical droplet size and oil/protein content. The emulsions with protein nanoparticles (pH 3) had a storage modulus ($G^{\prime }$) that was twice as high (∼7 kPa) as emulsions without nanoparticles (pH 7) (∼3.6 kPa) (Figure 1c). The emulsions behaved as solid‐like elastic systems, as $G^{\prime }$ was higher than $G^{\prime \prime }$ over a wide frequency range, regardless of pH or the presence of protein nanoparticles (Figure S2). Both $G^{\prime }$ and $G^{\prime \prime }$ increased slightly with increasing frequency and the loss tangent (δ) remained largely unchanged. The differences observed between the emulsions are attributed to protein conformation rather than changes in the physicochemical properties of the proteins. Protein conformation is the main determining factor in emulsion mechanical properties as it dictates droplet size and stability against coalescence and creaming. By changing the pH, the balance between repulsive electrostatic forces and attractive van der Waals and hydrophobic forces shifts, directly influencing protein conformation. For example, reducing the protein dispersion pH to 6.0 brings the repulsive and attractive forces in balance, causing liquid‐liquid separation [33]. Further pH reduction tips the balance in favor of attractive forces and extensive protein aggregation, yielding poor emulsifying capacity. Acidification past the isoelectric point reintroduces electrostatic repulsion, and depending on the time spent near the isoelectric point, protein aggregation may be limited. With rapid acidification to pH 3, only small protein particles are produced and many monomeric proteins also remain in dispersion [17]. Due to the abundance of monomeric proteins at pH 3, similar to pH 7, nearly the same droplet size is produced as with exclusively monomeric proteins at pH 7. As evident in Figure S3, there is a strong negative correlation between emulsifying capacity of the protein dispersion, given as droplet size ($D_{3,2}$), and emulsion mechanical properties ($G^{\prime }$). However, despite the similar droplet size, the emulsion at pH 3 exceeds the mechanical strength of the emulsion at pH 7. This indicates that, the protein nanoparticles must influence the emulsion mechanical properties in some other way than reducing droplet size.

### 2.2 Droplet Linking in Flow Using the Protein Nanoparticles

The ability of protein nanoparticles to form shear‐resistant interdroplet links is essential for designing materials that require delicate material responses. The formation of protein nanoparticles and the subsequent linking when droplets are in contact are probably related to the surface potential. To validate the hypothesis regarding the effect of charge on droplet linking by protein nanoparticles, we used on‐chip microfluidics for microscopic visualization of droplet interactions. Diluted emulsions, formed in either pH 3 or 7, flowed freely through the microfluidic channels and no aggregation could be observed (Figures S4 and S5). The dispersed nature of both emulsions shows that limited attractive interactions were present between droplets. These interactions were likely limited due to the high surface potential of the proteins and the low concentration of the droplets, which did not allow the droplets to come close enough together (Figure S6).

To understand the effect of electrostatic interactions on the droplet linking, we screened protein charges by co‐flowing the nanoparticle emulsion (pH 3) with a NaCl solution. Due to laminar flow in the microfluidic channel, there was initially limited mixing of the emulsion with the NaCl solution (Figure 2a‐I), but when the flow was arrested, salt ions instantaneously  diffused toward the oil droplets. As shown in Figure 2a‐II, III, after screening the system's charges with salt ions, droplet aggregation was observed, as a result of reduced electrostatic repulsion. When the electrostatic charges were sufficiently screened, weakly attractive hydrophobic and van der Waals forces became dominant, allowing droplets to come into close contact. In Figure 2b, a zoomed‐in region of interest shows how droplets came together to form aggregates in the presence of nanoparticles directly after the flow was arrested. Once formed, the aggregated oil droplets at pH 3 (with nanoparticles) remained aggregated despite the prolonged and increasing flow rate, as can be seen in Figure 3a‐I–III. Even when a droplet aggregate of approximately 100 µm was pushed toward a narrow channel of 15 µm (Figure 3b‐I), the aggregate temporarily deformed to accommodate the size restriction (Figure 3b‐II) but remained largely intact (Figure 3b‐III).

**FIGURE 2:**
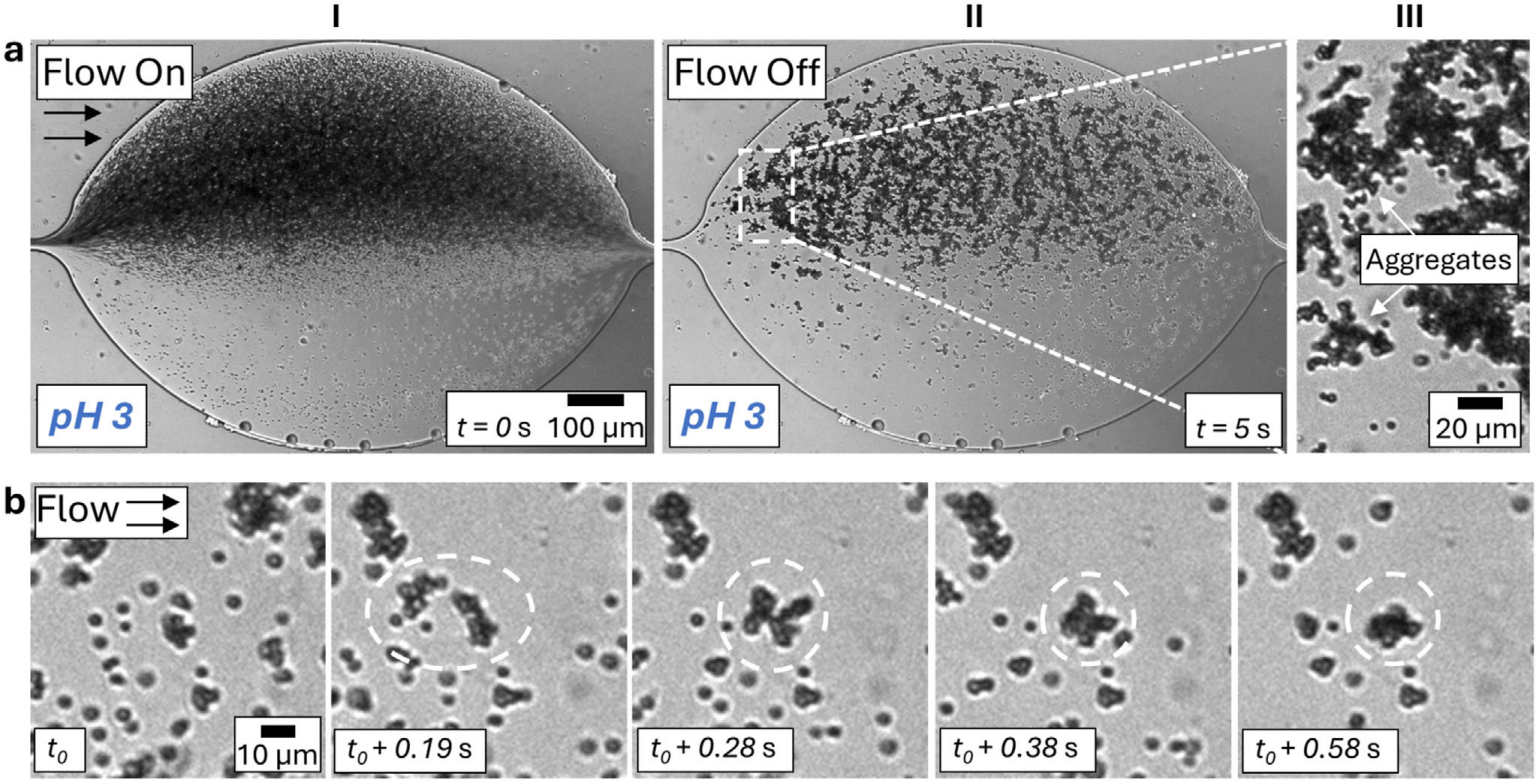
Real time aggregation of emulsion droplets decorated with nanoparticles in presence of ≈ 0.2 M NaCl at pH 3. (a‐I) NaCl solution and emulsions move through the chamber at a high flow rate without mixing, hence preventing aggregate formation. The applied pressure was 1000 mbar to both inlets. (a‐II) Upon arresting flow (applied pressure of 0 mbar), salt diffused through the chamber and the emulsion droplets aggregated due to the screened surface potential. (a‐III) Inset with a zoomed‐in view of the droplet aggregates. (b) Time lapse showing aggregation of emulsion droplets in the absence of applied pressure.

**FIGURE 3:**
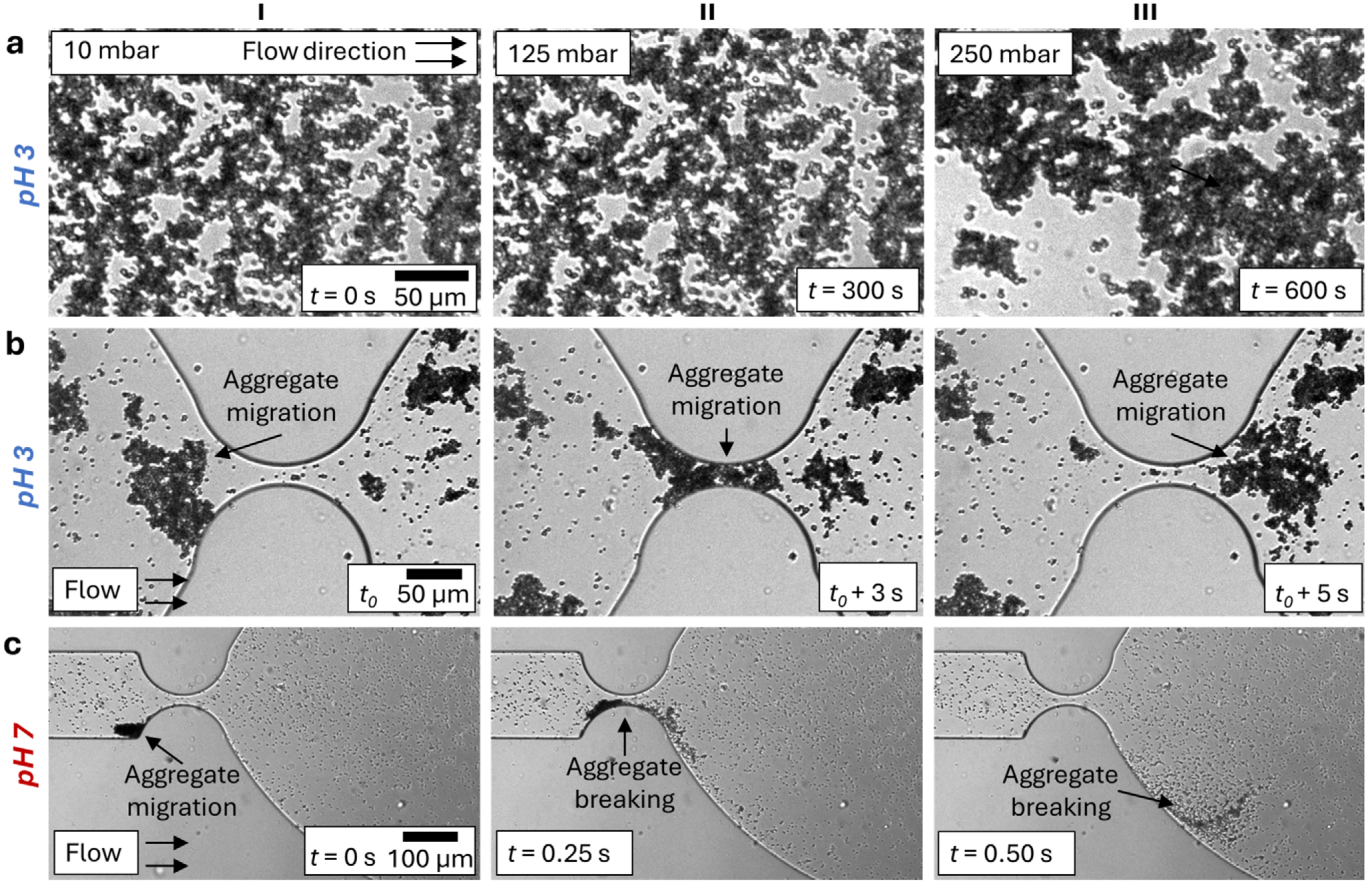
(a) Aggregates of droplets with nanoparticles exhibit negligible dissolution despite a continuously increasing flow of water. The applied pressure to the water channel started at 10 mbar (a‐I) and increased gradually to 125 mbar after 5 min (a‐II) and 250 mbar after 10 min (a‐III). (b) Droplet aggregates with nanoparticles and ≈ 0.2 M NaCl, once dislodged after application of a high water flow (250 mbar), are resistant to flow‐induced shear while migrating through a constriction. (c) Dissociation of droplet aggregates without nanoparticles (pH 7), under a low flow of water (10 mbar).

The same aggregation of oil droplets through charge‐screening was apparent at pH 7, despite the absence of the protein nanoparticles (Figure S7). However, in contrast to the shear‐resistant interdroplet links formed in the presence of the nanoparticles, the aggregated droplets without nanoparticles (pH 7) broke up into individual droplets by a minor flow increase. Aggregate dissociation occurred readily as the secondary inlet was changed from a NaCl solution to ultra‐pure water, even at a low flow rate (corresponding to approximately 10 mbar). The dissociation of a droplet aggregate is shown in Figure 3c. Initially, the flow starts to lift and push the droplet aggregate towards a narrow gap in the channel (Figure 3c‐I), where the aggregate breaks apart (Figure 3c‐II), and then breaks up into individual droplets as the aggregate passes the narrow gap (Figure 3c‐III). This dissociation behavior suggests that the droplets at pH 7 (without nanoparticles) were only weakly attractive, but not strongly adhered, as they dissociated easily when the flow was increased. Video recordings of the droplet aggregate behavior with and without the protein nanoparticles can be found in Videos S1 and S2.

The difference in resilience of the interdroplet links verifies our hypothesis that a strong adhesive force must be present between the oil droplets with nanoparticles (pH 3). Raising the ionic strength reduced electrostatic repulsion between droplets allowing them to approach to such an extent that nanoparticles could adhere to the surface of neighboring droplets, thus irreversibly linking them together. This is a clear indication that for nanoparticles to provide an adhesive effect, the interdroplet distance must be reduced to the point that the nanoparticles can link multiple droplets; the attractive force must overcome the electrostatic repulsion between the droplets.

### 2.3 Interfacial Rheology

#### 2.3.1 Single Interfaces

Interfacial rheology can shed additional light on droplet‐droplet interactions by probing lateral interactions among adsorbed proteins. To test whether the conditions driving interdroplet linking in flow coincide with lateral interactions between nanoparticles and other interfacial components, we studied an oil–water interface during cycles of compression and expansion. Changes in interfacial tension during cycles of deformation provide information on the dilatational storage modulus ($E_{d}^{\prime }$) and dilatational loss modulus ($E_{d}^{\prime \prime }$) of the interface. Again, an interface with a monomeric protein‐particle mixture (pH 3) was compared to an interface without nanoparticles (pH 7).

The evolution of dilatational moduli for the two interfaces (pH 3 and 7) over a range of interfacial deformations, as shown in Figure 4a, indicates that both interfaces were predominantly elastic ($E_{d}^{\prime }>E_{d}^{\prime \prime }$), and generally similar albeit being slightly stronger for pH 7. Clearly, under these conditions, the interactions between single proteins contributed more to the $E_{d}^{\prime }$ of the interface than the nanoparticles. By plotting the normalized interfacial tension versus the change in the interfacial area, we obtained cyclical Lissajous–Bowditch curves, which reflect the interfacial changes within cycles of deformation, as shown in Figure 4b. The similarity of these intracycle curves also highlight the similar behavior between the two probed interfaces. At higher deformations, the non‐linear and asymmetric response (third and fourth panel of Figure 4b) showed minor strain hardening upon compression as indicated by a nearly straight line from ultimate expansion to ultimate compression. No difference was observed between interfaces with or without protein nanoparticles, showing the dominant role of the individual proteins on the interface.

**FIGURE 4:**
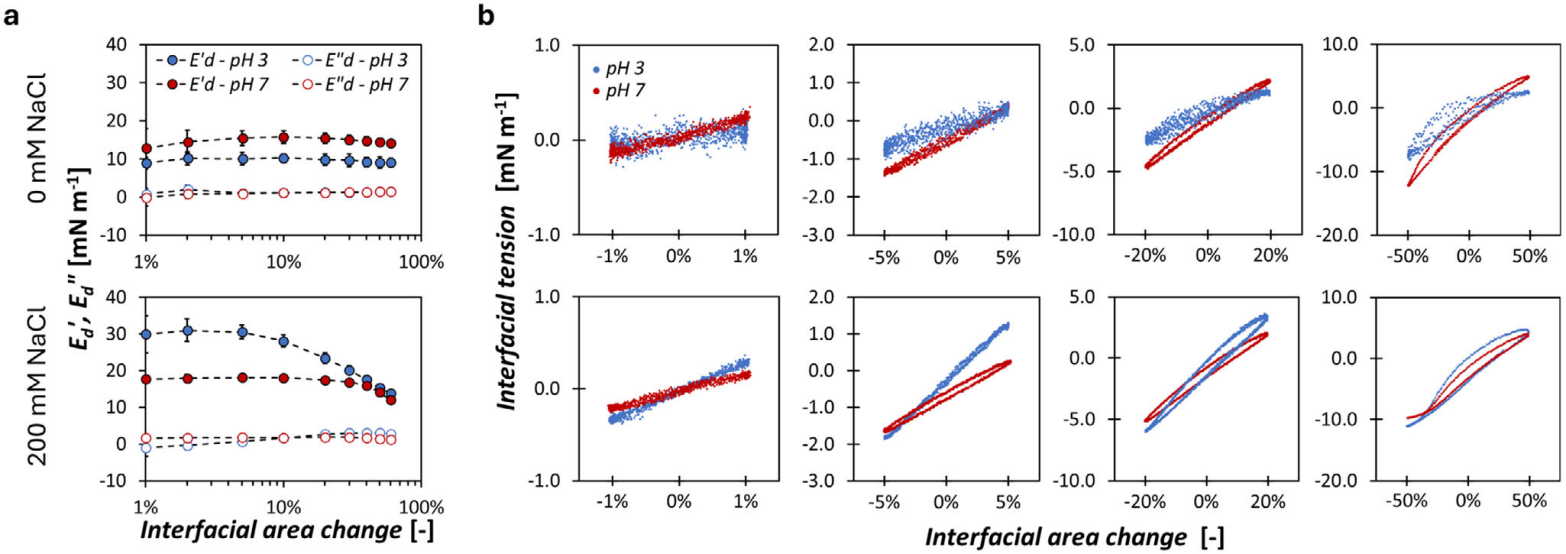
Rheological behavior of interfaces with (pH 3) and without (pH 7) nanoparticles in (top) without addition of NaCl and (bottom) with 200 mM NaCl. (a) Dilatational moduli of the films over a range of interfacial area deformation. (b) Lissajous‐Bowditch curves showing intracycle behavior at a selection of deformations.

To understand the effect of the protein nanoparticles at high ionic strength (200 mM NaCl), we also measured the interfacial dilatational moduli of the interfaces under these conditions. The increase in ionic strength resulted in an increase in $E_{d}^{\prime }$ for interfaces with nanoparticles (pH 3) and without (pH 7), but more drastically for the interface with nanoparticles (from ∼ 10 to ∼ 30 mN m^−1^ as opposed to from ∼ 15 to ∼ 18 mN m^−1^) (Figure 4a, bottom). The discrepancy between the increase in $E_{d}^{\prime }$ for interfaces with and without nanoparticles is likely a result of stronger lateral interactions between nanoparticles and individual proteins. Still, this discrepancy diminished with higher deformations, due to breakdown of the interfacial microstructure. Since larger particles (nanoparticles as opposed to individual proteins) typically have a high adsorption energy ($\gg k_{B}T$) [20, 21], the observed breakdown is instead a result of yielding, where the lateral interactions on the interface cannot withstand the deformation and the interface begins to flow. The intracycle behavior confirms the greater $E_{d}^{\prime }$ at low deformations, but the difference diminished at deformations ≥50% (Figure 4b, bottom). Furthermore, at high deformations, a distinctive tail near ultimate compression can be seen (Figure 4b, bottom‐fourth panel), indicating a breaking point in the interfacial microstructure. However, since the interface without nanoparticles exhibits the same behavior, the tail must be a consequence of individual proteins being expelled from the interface rather than the nanoparticles [34, 35].

Overall, the formation of shear‐resistant links between droplets as a result of charge screening coincided with an increase in $E_{d}^{\prime }$, potentially due to promoted lateral interactions between nanoparticles and individual proteins. However, since these lateral interactions yielded already at low deformations and were also observed when no particles were present, it is questionable whether the adhesion between droplets is driven by such lateral interactions.

#### 2.3.2 Thin Film Dynamics

To probe the adhesive interactions between opposing interfaces, a pressure profile mimicking the approach and separation of two droplets was used in a dynamic thin film balance [36, 37, 38, 39]. Here, two interfaces were first forced together with an external pressure reducing the film thickness from ∼ 1 µm to local thicknesses of 10 to a few 100 nm, matching the diameter of individual protein nanoparticles. Then, an opposing pressure was applied to separate the droplets again, thus mimicking the approach and separation of two droplets in an emulsion. By standardizing the time before pressure reversal, the expansion and retraction rates of the changes in the radii of the thin films with and without nanoparticles were compared. Both the morphology obtained via interferometry during the approach and separation phases and the rate of retraction during separation were used to gain insight into the adhesion between droplets.

Upon application of a positive pressure from the outer (oil) phase on the thin film, the pressure balance between the film and Plateau border shifts such that liquid starts to drain from the film, causing the thin film radius (R) to expand, called the approach phase. At $R=R_{max}$, further expansion of the thin film is halted because the disjoining pressure has restored the pressure balance between the film and Plateau border thus preventing further drainage [40]. The separation phase, initiated by subsequent inversion of the applied pressure, is the filling of the thin film from the Plateau border leading to retraction of the thin film. The normalized thin film radius versus the normalized time of duplicate pressure step measurements of films with protein nanoparticles (pH 3) and without protein nanoparticles (pH 7) is shown in Figure 5a. The approach phase for films with nanoparticles (pH 3) and films without nanoparticles (pH 7) followed the same general trend with only small deviations, potentially due to minor differences in interfacial or bulk viscosity. The separation phase, initiated by subsequent inversion of the applied pressure, however, showed drastically different behavior between the films with (pH 3) and without nanoparticles (pH 7). For the film without nanoparticles, pressure inversion led to a more rapid filling of the thin film and consequent retraction of R. The retraction rate of R during separation was ∼4× higher than the expansion rate during the approach phase. The discrepancy between expansion and retraction rates, despite the equal pressure difference between the approach and separation phases, suggests an additional force driving the separation of the two interfaces without nanoparticles. This additional force is caused by changes in the local curvature of the droplets as they flatten where the droplets meet during the formation of a thin film. The change in local curvature introduces a stress on the system [3, 38] and gives rise to forces such as disjoining pressure, hydrodynamic pressure and capillary forces [39, 41]. In contrast, the retraction rate of R for the film with nanoparticles is roughly equal to the expansion rate during the approach phase. The reduced retraction rate, relative to that of the film without nanoparticles, is a clear indication of stronger attractive forces that hold the interfaces together and prevent liquid flow from the Plateau border into the film. These stronger attractive forces are responsible for the limited droplet‐droplet separation, i.e., enhanced adhesion.

**FIGURE 5:**
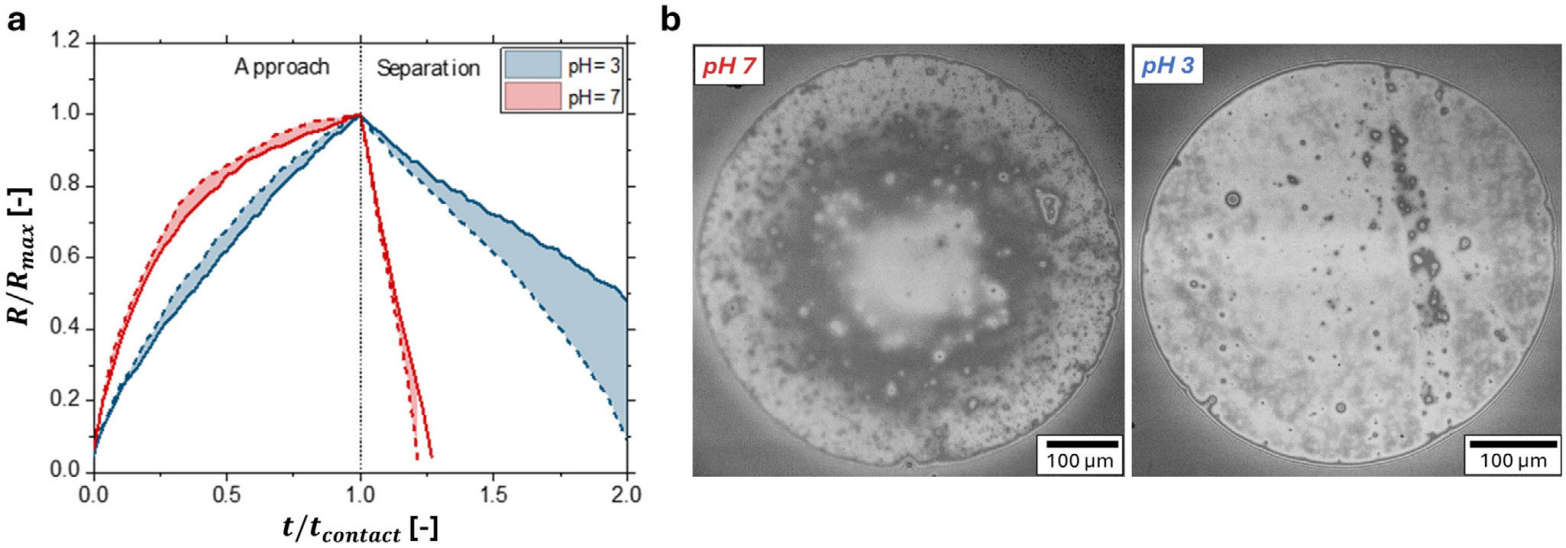
Thin liquid film morphology and evolution. (a) Evolution of the normalized thin liquid film radius ($R/R_{max}$) plotted versus the time normalized over the contact time ($t/t_{contact}$) during the alternating positive/negative pressure step cycle. (b) Morphology of the thin film at $R=R_{max}$ with film thickness indicated by alternating light and dark rings obtained from interferometry (scale bar = 100 µm).

Insights into the mechanism of adhesion can be gained from the morphology of the thin film, obtained from the interference patterns. For the interface with nanoparticles (pH 3), the film was planar with a predominant thickness of 9 nm in co‐existence with thicker local regions of ≈ 100–500 nm (Figure 5b). In these thicker regions, the distance between the two interfaces is in the same range as the diameter of single nanoparticles (30‐450 nm, after filtration), suggesting that individual nanoparticles trapped between the interfaces locally deformed the film. This morphology was in stark contrast with that of the film without nanoparticles (pH 7), which was dimpled, i.e. a maximum thickness was in the center of the film and a minimum thickness along the edge. The lack of such a dimple for the film with nanoparticles indicates adhesion between the opposing interfaces upon initial close contact at small R, thereby preventing the interfaces from separating again as the thin film expanded. While the interference patterns provide information on the local film thickness, the microscopic image also shows the film area affected by these nanoparticles, which was much larger than the size of individual nanoparticles. This could be because the interface was not tightly wrapped around the nanoparticles, but draped more loosely over them, thereby minimizing the total interfacial area. Alternatively, instead of single particles, clusters of aggregated nanoparticles might be present in a 2D‐plane, due to possible capillary attraction between the nanoparticles [26, 28, 42, 43]. The differences in film morphology (dimpled versus planar) and film retraction rates suggest that the adhesive forces observed during separation originate from bridging interactions, i.e., nanoparticles adhering to both opposing interfaces. Video recordings of the thin film growth and retraction are given in Video S3 (pH 3) and Video S4 (pH 7).

Despite the low ionic strength, there were clear signs of adhesion between the nanoparticle‐laden interfaces, in contrast with the results obtained by the previous experiments. This is most likely related to the ability of the oil droplets to approach each other. In the flow experiments, the nanoparticles were adsorbed on one interface, and could not come into close enough proximity with other oil droplets to bridge them. Only when the electrostatic repulsion between droplets was reduced through charge screening, such droplet linking was observed. Contrarily, in the thin film experiments, the applied external pressure forced the interfaces within distances smaller than the nanoparticle diameter, allowing particle‐bridging to occur more rapidly.

In addition to low ionic strength, the thin film experiment was also performed with 200 mM NaCl. However, extensive protein aggregation led to highly heterogeneous films and channel clogging, making it impossible to assess these films quantitatively. Still, under these conditions, signs of strong attractive intermolecular forces between the interfaces are still evident from the pinning of the interfaces during film separation. Instead of an even radial retraction of the thin film, some regions of the film were “pinned,” i.e. stuck together, resulting in a delay in the separation of the two interfaces, and a temporary deviation from circularity. Pinning of the interfaces was apparent for films at both pH values. Video recordings of the thin film expansion and retraction are given in Video S5 (pH 3) and Video S6 (pH 7).

### 2.4 Modeling the Contribution of Adhesive Interactions in the Film

Next to the adhesive interaction observed between opposing films in the thin film balance, a secondary indication for this adhesive interaction could be obtained by measuring the contact angle between linked droplets [2]. Non‐zero contact angles were measured for emulsions with nanoparticles (pH 3) at low and high ionic strength ($\theta_{\mathrm{conf}}=$ 39.0±7.6° at low ionic strength, $\theta_{\mathrm{conf}}=$ 47.2±5.5° at high ionic strength), and for emulsions without nanoparticles (pH 7) at high ionic strength ($\theta_{\mathrm{conf}}=$ 31.5±6.4). No linked droplets could be observed in emulsions without nanoparticles (pH 7) at low ionic strength. The contact angle between linked droplets can be related to an adhesive force between the opposing interfaces. Assuming that a thin liquid film can be described as two flat interfaces at a distance h apart, the $\Pi_{disj}\left(h\right)$ is determined by the sum of forces acting on the film as a function of h. This dependence on h is described for van der Waals and electrostatic forces by the Derjaguin, Landau, Verwey, and Overbeek (DLVO) theory [44, 45], but also holds true for non‐DLVO forces like steric and bridging interactions [46, 47, 48]. For adhesive interfaces, $\Pi_{disj}\left(h\right)$ is negative at a long distance, but becomes positive at a shorter distance, preventing coalescence. At an equilibrium distance, $h_{e}$, the interfaces do not attract or repel each other. The resulting work of adhesion at this point, $E_{adh}$, is given by [49]:

(1) $$E_{adh}=E\left(h_{e}\right)=-\int_{\infty }^{h_{e}}\Pi_{disj}\left(h\right)\;dh$$

This $E_{adh}$ can be related to the surface energy of the film ($\sigma_{f}$), as the surface energy is related to h and $\Pi_{disj}$. For large h, when droplets are too far apart for attractive forces to affect them, E(h)= 0 and $\sigma_{f}=2\sigma_{int}$, where $\sigma_{\mathrm{int}}$ is the interfacial tension of a single interface. However, at small h, the formation of a thin film and deformation of the droplets reduces the surface energy. At equilibrium distance ($h_{e}$), the surface energy of the film is reduced by the (negative) energy of adhesion as:

(2) $$\sigma_{f}=2\sigma_{int}+E\left(h_{e}\right)$$

Since the tension of the film is different from that of two isolated interfaces, a contact angle, $\theta_{adh}$, is expected at the junction between connected interfaces, as shown in Figure S8. The contact angle is related to the surface energy by:

(3) $$\sigma_{f}=2\sigma_{int}cos\left(\theta_{adh}\right)$$

By combining these equations, we obtain a relation between the contact angle ($\theta_{adh}$) and the adhesion energy ($E_{adh}$), known as the Young–Dupré equation:

(4) $$E_{adh}=2\sigma_{int}\left[1-cos\left(\theta_{adh}\right)\right]$$

which relates to the disjoining pressure as follows:

(5) $$E_{adh}=E\left(h_{e}\right)=-\int_{\infty }^{h_{e}}\Pi_{disj}\left(h\right)\;dh=2\sigma_{int}\left[1-cos\left(\theta_{adh}\right)\right]$$

Equation (5) allows a rough comparison between the experimentally measured contact angles and those theoretically estimated from the disjoining pressure. The disjoining pressure ($\Pi_{disj}$) is taken as the sum of van der Waals ($\Pi_{vW}$), electrostatic ($\Pi_{el}$), steric ($\Pi_{st}$), and the attractive bridging interactions ($\Pi_{br}$) as:

(6) $$\Pi_{disj}\left(h\right)=\Pi_{vW}\left(h\right)+\Pi_{el}\left(h\right)+\Pi_{st}\left(h\right)+\Pi_{br}\left(h\right)$$

The van der Waals interactions give an attractive force at short distances:

(7) $$\Pi_{vW}=-\frac{A_{H}}{6\pi h^{3}}$$

with $A_{H}=$8.12×10^−21^ J calculated from the Lifshitz theory [46] using a refractive index of 1.474, and a dielectric constant of 3.2 for rapeseed oil, as reported in literature [50, 51]. The electrostatic contribution is given by [52]:

(8) $$\Pi_{el}=64C_{el}kTtanh^{2}\left(\frac{e\Psi_{s}}{4kT}\right)exp\left(-\kappa h\right)$$

where $C_{el}$ is the electrolyte concentration, $\Psi_{s}$ is the surface potential, and κ is the Debye screening length. The steric interactions are described by [46]:

(9) $$\Pi_{st}=A\frac{kT}{\lambda_{st}}\frac{h}{\lambda_{st}}exp\left(-\frac{h}{\lambda_{st}}\right)$$

where A is a prefactor that describes the surface concentration of protrusions (fitting parameter) and $\lambda_{st}$ is the decay length as described in Ref. [53]. Finally, the bridging attraction can be described by:

(10) $$\Pi_{br}=\frac{BkT}{\lambda_{br}-h}$$

for $h>\lambda_{br}$ [47, 48], where B is a prefactor that describes the surface concentration of bridging sites (fitting parameter), and $\lambda_{br}$ is the decay length. With these equations we can obtain order of magnitude estimates of the different effects at play in the studied emulsions.

Using experimental values for the surface potential ($\Psi_{s}$) and electrolyte concentration ($C_{el}$) at low ionic strength, where the surface potential is greatest, still the electrostatic interactions become negligible for film thicknesses above 10 nm. This indicates that the interfaces with nanoparticles cannot approach each other to a distance h short enough for electrostatic forces to have an effect on the disjoining pressure. The electrostatic repulsion obtained from the nanoparticles protruding from the interface was assumed negligible due to their limited coverage (≈ 3%) of the interface [17]. Two contributions to the overall steric disjoining pressure were considered, assuming that the total steric interactions are the sum of protein nanoparticles and monomeric proteins. A $\lambda_{st}$ of 9 nm was chosen, assuming that the protrusions in the thin regions of the film are located over a protein monolayer, similar to Ref. [54], and that the monolayer has a size equal to that of a pea protein molecule, which has been estimated to be 4.5 nm [55, 56]. For thicker regions containing nanoparticles, a $\lambda_{st}$ equal to 200 nm was chosen based on the measured particle size distribution (Figure 1a). Using these values as fixed constants, the A parameter was obtained using the Least Squares method (OriginPro, Levenberg‐Marquardt (L‐M) Algorithm) with the extra constraint to achieve a maximum critical pressure of 60 kPa, which is equal to the average Laplace pressure in the emulsions, calculated using the experimentally determined droplet size (Figure S1) and interfacial tension values (25.5 and 20.2 mN/m, for pH 3 and 7, respectively). The obtained value for A for both situations was ≃ 3×10^17^ m^−2^. This value is of the same order of magnitude as the C constants obtained for lecithin in Ref. [53] after assuming that the force in our experiments is $∼\Pi_{disj}\pi R_{f}^{2}$ [57] with the film radius $R_{f}$ being O(10^−4^) m (Figure 5b). Since the contribution of $\Pi_{vW}$ and $\Pi_{el}$ only become apparent for film thickness h smaller than the nanoparticle diameter, the only remaining terms for the disjoining pressure are the steric ($\Pi_{st}$) and bridging interactions ($\Pi_{br}$). For the bridging interactions, the $\lambda_{br}$ was taken as 200 nm, using the experimentally determined particle size distribution (Figure 1a). Using this value as a fixed parameter, the value for B was obtained using the same fitting procedure as mentioned earlier, using a maximum value for $\Pi_{br}$ that is equal to the maximum value of $\Pi_{st}$ at h= 200 nm (to ensure continuity in the overall disjoining pressure). This approach resulted in a value for B of 2 × 10^31^ m^−2^. It should be noted that, for the many reasons stated in Ref. [48], the model for bridging interactions is very approximate and is used here to provide estimates for the adhesive forces rather than to interpret the actual physical mechanism involved in the attraction between particles.

The estimated disjoining pressure was plotted over film thickness to asses whether the theoretical model aligns with the experimental thin films. Two scenarios were considered, i) pure protein interfaces with a film thickness of 9 nm, and ii) interfaces with large aggregates (nanoparticles of 200 nm), giving distinct profiles of disjoining pressure (Figure 6). Here, it is evident that for interfaces with exclusively individual proteins on the interface, as h approaches $h_{e}$ at ∼ 10 nm, $\Pi_{st}$ increases the $\Pi_{disj}$ to a maximum. This theoretical equilibrium thickness $h_{e}∼$ 10 nm is approximately equal to 2 times the experimentally observed thickness of interfacial pea proteins [55]. For $h<h_{e}$, attractive $\Pi_{vW}$ reduces $\Pi_{disj}$ until coalescence. In contrast, for films with a mixture of nanoparticles and individual proteins, at h>200 nm (particle size), $\Pi_{disj}$ is low but increases to a local maximum at 200 nm due to steric interactions between the nanoparticles and the opposing interface. At h< 200 nm, there are no nanoparticles and $\Pi_{disj}$ becomes the same as that of a thin film without nanoparticles. The area under the curve of each scenario gives an indication of the difference in magnitude of the film contribution to bulk emulsion properties. Since the area for the system with protein nanoparticles is an order of magnitude greater than that of the system without nanoparticles, it is evident that this contribution cannot be ignored. Furthermore, this difference in film contribution is in agreement with the measured difference in thin film retraction rate during separation ($\frac{dR^{*}}{\mathrm{dt}}=$ 0.0163 s^−1^ with nanoparticles, as opposed to 0.123 s^−1^ without nanoparticles). The contact angle given by the theoretical disjoining pressure obtained from Equations (5) and (6) is $\theta_{\mathrm{disj}}\approx$ 45°. If instead the long‐range bridging interactions from the thin film are ignored, a zero contact angle is predicted, i.e., the droplets are non‐adhesive. This clearly demonstrates the crucial role of these bridging interactions by nanoparticles to the net attractive force between droplets resulting in their adhesion. Contact angles were determined from confocal micrographs to check the validity of the theoretical contact angles. The emulsion with nanoparticles at high ionic strength (pH 3 ‐ 200 mM NaCl) had contact angles of 47.2±5.5°, very similar to the theoretical prediction. Without nanoparticles and at low ionic strength (pH 7 ‐ 0 mM NaCl), no contact angles could be measured, meaning the droplets were non‐adhesive, as expected. Increasing the ionic strength (pH 7 ‐ 200 mM NaCl) caused droplet flocculation and gave smaller contact angles (35.1±6.4°). Similarly, for the emulsion with nanoparticles and low ionic strength (pH 3 ‐ 0 mM NaCl), the contact angles were on average lower than at high ionic strength (39.0±7.6°). The reduced contact angles at low ionic strength and without nanoparticles indicate the difference in adhesion energy between simple flocculation and nanoparticle‐mediated adhesion.

**FIGURE 6:**
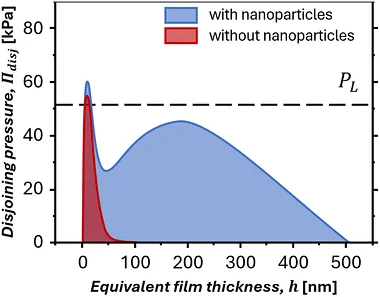
Disjoining pressure ($\Pi_{disj}$) as a function of film thickness (h) in two scenarios, i) a thin film with exclusively individual proteins on the interfaces, or ii) a thin film with a mixture of individual proteins and adhesive nanoparticles. The dashed line shows the Laplace pressure ($P_{L}$) for comparison.

### 2.5 Proposed Mechanism and Proof of Concept

Combining the gained knowledge from the previous experiments and theoretical model, we propose a mechanism for the ion‐induced nanoparticle‐mediated interdroplet linking. Initially, oil droplets are enveloped by a layer of individual proteins and a limited number of protein nanoparticles. The nanoparticles are partly present on the interface, but a large fraction most likely also remains in the continuous phase. When ionic strength is increased by adding salt, the salt ions screen the surface potential of the proteins, thereby reducing electrostatic repulsion. Due to the reduced electrostatic repulsion, the nanoparticles in the continuous phase can now more easily adsorb to droplet interfaces. The adsorption of more nanoparticles provides the droplets with more bridging sites. When two droplets approach, either through random flow, applied shear, or an external pressure as in the thin film experiments, the protruding nanoparticles are able to link two droplet interfaces by attractive bridging (Figure 7). High ionic strength aids in the bridging interaction between droplets by reducing the repulsive electrostatic force, allowing droplets to come close enough together to form links (Figure 2b). The presence of a nanoparticle at two interfaces simultaneously leads to strong links between the droplets. Through the formation of these shear‐resistant links, the bulk emulsion is granted mechanical strength.

**FIGURE 7:**
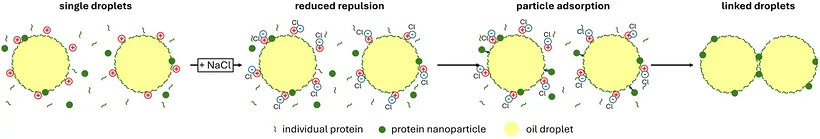
Proposed mechanism for interdroplet linking mediated by nanoparticles and triggered by addition of salt.

To evaluate the effect of interdroplet linking on bulk emulsion mechanical properties, emulsions were prepared with nanoparticles (pH 3) and without (pH 7) with identical oil volume fraction ($\phi_{\mathrm{oil}}=$ 0.74) over a range of ionic strength values (0, 50, 100, and 200 mM NaCl) in the continuous phase (Figure 8a). Without additional salt, the emulsion with nanoparticles (pH 3) has a higher $G^{\prime }$ than the emulsion at pH 7, as was already observed in Figure 1c. Upon addition of salt, the emulsion at pH 3 showed an increase in $G^{\prime }$ from ∼ 1.9 to ∼ 4.6 kPa at 200 mM NaCl. The beneficial effect of NaCl was already evident at concentrations as low as 50 mM, but appeared to diminish between 100 and 200 mM. Therefore, the range of NaCl concentrations expected to be beneficial for the emulsion's mechanical strength lies between 0 and 200 mM, with only limited benefits above 200 mM. Concentrations below 50 mM likely still have a minor beneficial effect to mechanical strength by screening charges and promoting droplet‐droplet contact. In contrast to the emulsion at pH 3, the emulsion at pH 7 increased only from 1.2 to 1.9 kPa between 0 and 200 mM NaCl. The changes in mechanical strength cannot be attributed to simple protein aggregation, as the NaCl concentration was within the salting in range (0.0–0.2 M NaCl) and well below the salting out concentration (≥0.4) [58]. Furthermore, at pH 7, the negatively charged proteins would interact primarily with Na^+^, which is a mild kosmotrope [59, 60]. Therefore, at this low NaCl concentration, the Na^+^ ions aid in the solubility of the proteins at pH 7. Evidence of a salting in effect at pH 7 can be seen in Figure S9, where larger particle sizes (peaks corresponding to 0.6–3.0 and 3.0–7.0 µm) were measured in absence of NaCl than in the case of 200 mM of NaCl. However, despite the protein aggregates formed at pH 7 and 200 mM NaCl being similar in size (50–900 nm) to the protein nanoparticles at pH 3, the NaCl concentration had a minimal effect on the mechanical strength of the emulsion at pH 7, in contrast to the emulsion at pH 3.

**FIGURE 8:**
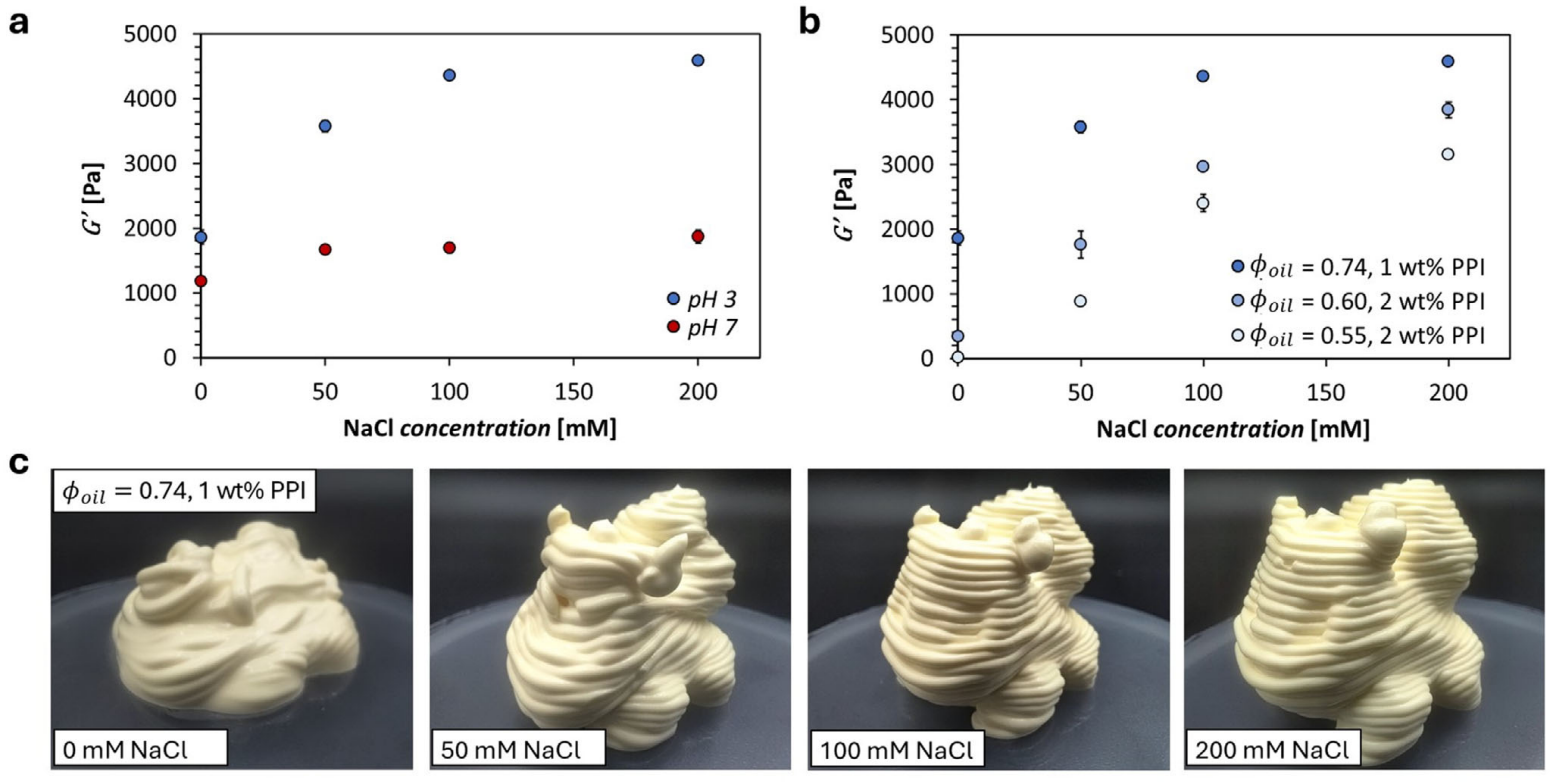
Mechanical transformation of emulsions via screening of surface potential. (a) Pronounced increase in storage modulus ($G^{\prime }$) for emulsions with adhesive nanoparticles (pH 3) compared to minor changes without adhesive nanoparticles (pH 7). The emulsions were made with 1 wt% protein and had an oil volume fraction of 0.74. (b) Effect of $\phi_{oil}$ and quantity of adhesive particles on bulk rheology. (c) 3D‐printed Pokémon that holds its shape progressively better with increased salt concentration.

To show that the increase in mechanical strength at pH 3 is a consequence of interdroplet linking, and thus proportional to the number of contact points between emulsion droplets, bulk rheological measurements were repeated for more dilute emulsions with twice the protein content (Figure 8b). The emulsions were below the random packing fraction ($\phi_{\mathrm{oil}}<$ 0.64) and had the same droplet size as the emulsions discussed before. Here, we observed that the emulsions with $\phi_{oil}$ of 0.55 and 0.60 were liquid‐like with $G^{\prime }$ in the order of ≤ 200 Pa at 0 mM NaCl. However, with addition of NaCl, the emulsions increased drastically in $G^{\prime }$, reaching a similar mechanical strength as the emulsions with $\phi_{\mathrm{oil}}=$ 0.74. The oil volume fraction has a direct impact on the emulsion mechanical properties as shown in Figure S10. Without droplet linking, the emulsion is solid‐like for oil volume fractions above 0.6 and liquid‐like below 0.6, as there is a steep increase in interfacial and electrostatic free energy contributions to the system when the volume fraction approaches the critical volume fraction [61]. However, by linking droplets, the emulsion already became solid‐like at much lower oil volume fractions (above 0.4), likely due to formation of linked droplet clusters, and consequent reduction of the effective jamming volume fraction. This behavior is similar to attractive emulsions [15]. The more than 170‐fold increase in $G^{\prime }$ for the emulsion with $\phi_{\mathrm{oil}}=$ 0.55 (from 18.5 to 3159 Pa) clearly illustrates the extent to which interdroplet linking can affect the mechanical properties of an emulsion. It also shows that by utilizing this mechanism, even more dilute emulsions may be created that still reach a $G^{\prime }$ similar to that of the extracellular matrix of animal fat tissue (3–5 kPa) [62].

To visualize the extent of mechanical changes by ion‐induced interdroplet linking, emulsions with nanoparticles at a $\phi_{oil}$ of 0.74 were 3D‐printed following the intricate geometrical design of a Pokémon, famous from the 90's television show and video games (Pokémon, Nintendo, Japan) (Figure 8c). Without NaCl, the Pokémon collapsed under its own weight (first panel). At slightly increased NaCl concentrations (50 mM), the emulsion was already much more able to support its own weight but still sagged considerably (second panel). From NaCl concentrations of ≥ 100 mM, the Pokémon had sufficient strength to support its own weight and improved in print fidelity. Shape retention of the samples at higher NaCl concentration was excellent as evident from the pictures shown in Figure 8c, which were made 1 day after printing. After two weeks of storage, water evaporated from the samples and subsequent oil leakage was observed. However, the geometry was unaffected by the evaporation and oil leakage. An example of the good shape retention despite water evaporation is given for an extruded emulsion in Figure S11. The rheological properties of the prints also did not change after 1 week, again confirming that the structure of the emulsions did not change. The good shape retention for these systems was also highlighted in the work of Sridharan [16]. These findings highlight the impact particle‐mediated adhesion may have in providing mechanical strength to emulsions.

## 3 Conclusions

The aim of this work was to understand the effect of protein nanoparticles in providing a tunable mechanical strength to emulsions. Driven by ionic strength, pea proteins self‐assemble into nanoparticles, which were able to link neighboring oil droplets together by strong attractive intermolecular interactions. These attractive interactions only lead to particle linking when neighboring droplets were able to get in close proximity to each other. The proximity between droplets was shown to depend strongly on the ionic strength. Increasing the ionic strength shifted the balance between attractive hydrophobic and van der Waals forces and repulsive electrostatic forces in favor of attraction, reducing the distance between oil droplets. Even in jammed systems, in which droplet mobility is already very limited, the ion‐induced formation of shear‐resistant interdroplet links provides the bulk emulsion with double the mechanical strength. In more dilute systems ($\phi_{oil}<\phi_{RCP}$), ion‐driven interdroplet linking increased $G^{\prime }$ by a factor 100–200. Experimentally determined contact angles of adhesive droplets also confirmed the synergistic effect of ionic strength on droplet adhesion. By exploiting experiments focused at different length scales (interfacial rheology, thin film dynamics, and bulk rheology), we were able to validate our proposed mechanism for interdroplet linking and verify the importance of bridging interactions in realizing an adhesive effect. Such an adhesive effect was obtained only when protein nanoparticles were present, which were situated at the interface of two droplets at the same time, thereby strongly linking the oil droplets. Utilizing such adhesive nanoparticles allows the creation of emulsions with tunable mechanical strength, transforming dilute liquid‐like emulsions into solid‐like emulsions. Notably, the formation of droplet‐bridges grants the required additional mechanical strength to jammed emulsions to accurately mimic the role of the extracellular matrix in animal fat tissue. Furthermore, we have shown that these emulsions fortified with interdroplet links possess the right characteristics to be 3D‐printed into intricate geometrical shapes, thus offering a promising perspective for their use in products where this is required, such as soft microelectronics [32].

## 4 Experimental Section

### 4.1 Materials

Dried yellow peas were purchased from Alimex BV (Sint‐Kruis, the Netherlands) and rapeseed oil was purchased from a local supermarket. All chemicals were obtained from Sigma Aldrich (Zwijndrecht, the Netherlands). All solutions, dispersions and emulsions were prepared using ultra‐pure water from a MilliQ filtration system.

### 4.2 Protein Extraction

Pea Protein Isolate (PPI) was obtained using an alkaline extraction method based on the work of Sridharan et al. [16]. First, the peas were dry‐milled in an IKA coffee mill (Staufen, Germany) and then soaked in water in a 10 wt% solids to water ratio. The pH of this dispersion was adjusted to 8 using 0.5 M NaOH under constant stirring (200 rpm) for 2 h, during which the pH was frequently adjusted to 8. This alkaline dispersion was then blended at maximum speed using a Waring CB15V 4‐liter blender (Waring, Torrington, CT, USA). The resulting slurry was centrifuged for 30 min at 10,000 g to precipitate solids. The protein‐rich supernatant was separated using a double cheesecloth and the pH of the filtrate was adjusted to 4.8 (the iso‐electric point) with 1 M HCl and left to stir (200 rpm) for 1 h during which the pH was frequently adjusted. The protein precipitate was then collected by centrifuging the solution again for 30 min at 10,000 g. The supernatant was discarded, and the pellet was redispersed in water in a solids to water ratio of 10 wt%. The pH of this protein dispersion was brought to 7 with 0.5 M NaOH and frozen (−18°C) for at least 24 h before freeze drying for 67 h at 0.01 mbar and −76°C . The resulting PPI powder was stored at −18°C until further use.

### 4.3 Physicochemical Characterization

#### 4.3.1 Protein Isolate Purity

The protein content of the pea protein isolate was determined using the Dumas method and a conversion factor of 5.7, as is common practice for legume proteins [33, 63, 64], giving a purity of 76.5 ± 1.9 wt%, similar to Ref. [17].

#### 4.3.2 Surface Potential

The surface potential of 0.01 wt% PPI dispersed in ultra‐pure water was measured using electrophoretic light scattering (Zetasizer Nano‐ZS, Malvern Ltd., UK). Before loading the sample into the capillary cell, the dispersion was sheared for 15 s at 8,000 rpm. The refractive index (n) and absorption (k) used for the measurements were 1.45 and 0.001, respectively. The samples were equilibrated for 120 s at 20°C prior to analysis. Afterwards, the dispersions were measured in triplicate.

#### 4.3.3 Particle Size

The particle size of PPI dispersions was measured using static light scattering (BetterSizer S3 Plus, BetterSize Instruments Ltd., Hamburg, Germany). To replicate the steps for emulsification, samples were sheared for 15 s at 8,000 rpm followed by 1 min at 10,000 rpm using an Ultra‐Turrax (IKA, Staufen, Germany), and finally passed through a high‐pressure homogenizer (Panda GEA Niro Soavi, Parma, Italy) for 5 cycles at 650 bar. The homogenized samples were measured in triplicate. The particle diameter was calculated as Sauter Mean Diameter ($D_{3,2}$) as follows:

(11) $$D_{3,2}=\frac{\sum x_{i}d_{i}^{3}}{\sum x_{i}d_{i}^{2}}$$

using the number percent ($x_{i}$) per particle size ($d_{i}$) as obtained via light scattering.

### 4.4 Emulsion Preparation and Characterization

Oil‐in‐water emulsions were prepared with PPI dispersed in ultra‐pure water. This dispersion was brought to pH 3 using 1 M HCl and left to disperse overnight at room temperature under constant stirring (300 rpm). The next day, the protein dispersion was sheared for 15 s at 8,000 rpm using a head‐stirrer (Ultra‐Turrax T25, IKA, Germany) to break up any large aggregates. Immediately thereafter, the lipid fraction was slowly poured into the PPI dispersion while applying high shear (10,000 rpm) for 1 min to form a coarse emulsion. The emulsions contained 1 wt% protein, ∼ 39 wt% water, and 60 wt% oil. The coarse emulsion formed after high‐shear homogenization was further homogenized using a high‐pressure homogenizer (Panda GEA Niro Soavi, Parma, Italy) for 5 cycles at 650 bar.

To determine the extent of inevitable dilution during loading of the high‐pressure homogenizer, the moisture content of the formed emulsions was measured using an HS153 Moisture Content Analyzer (Mettler Toledo, Tiel, the Netherlands). The device was programmed to heat the sample (2±0.2 g) to 105°C, while measuring the weight loss. The measurement ended when the rate of weight loss was lower than 1 mg per 50 s. Each sample was measured in triplicate. Afterwards, the emulsions were diluted to ensure all had the same oil volume fraction.

The droplet size distribution of the emulsion was determined using the same static light scattering technique as in Section 4.3.3. To ensure the measured sizes were of individual oil droplets and not flocculates, ∼ 0.2 g of emulsion was dispersed in 4 mL of 1 wt% sodium dodecyl sulfate (SDS), which displaces protein on the interface and disperses the flocculates with their high negative potential. SDS‐emulsion dispersions were left to stir (200 rpm) for 30 min before measuring. All samples were measured in triplicate.

### 4.5 Confocal Laser Scanning Microscopy

Emulsions were imaged using a ReScan confocal microscope (RCM 2.5, Confocal NL, the Netherlands) fitted with a Nikon Plan Apo 60×A/1.40 oil immersion lens (∞/0.17, WD 0.21, DIC H; Nikon, Europe B.V., Amstelveen, the Netherlands). Samples were loaded onto Epredia cover slips (24 × 60 mm # 1,5) and stained using a mastermix containing 100 µg mL^−1^ Rhodamine B for protein, and BODIPY 493/503 for lipids, in DMSO. Staining was done just before placing the cover slip on the inverted frame microscope (Nikon Eclipse Ti, Nikon Europe B.V., Amstelveen, the Netherlands) by applying a 10 µL drop of the mastermix to the periphery of the sample. This way, the dyes could diffuse into the emulsion while minimizing dilution. An Oxxius L4Cc laser (Oxxius, Lannion, France) with 488 and 561 nm was used for excitation and emission was detected using a Tucsen FL 20BW CCD camera (Tucsen, Fuzhou, China). The obtained micrographs were analyzed in Fiji (version 1.52).

The contact angle between linked droplets was determined using the Contact Angle plugin in Fiji. A schematic explaining the process is given in Supporting Information, Figure S8.

### 4.6 Bulk Shear Rheometry

Emulsions were prepared as described before, except that afterwards the NaCl concentration in the continuous phase was brought to 0, 50, 100, or 200 mM using a 2 M NaCl solution and ultra‐pure water to ensure equal dilution. The rheological properties of the emulsions were measured using a modular compact rheometer MCR 302 (Anton Paar, Graz, Austria) and a parallel plate (plate‐plate) setup with a diameter of 25 mm and a employed gap size of 1 mm. The plates were profiled in a cross‐hatch pattern to prevent wall slip (PP25/P2/SS, Anton Paar, Graz, Austria). The samples were sheared with an increasing strain ramping logarithmically from 0.01 to 1,000%, measuring each strain until steady state before moving to the next. The strain was applied through an oscillatory shear at a frequency of 1 Hz while maintaining a constant temperature of 20°C. All experiments began with a 5 min relaxation period to allow material relaxation after applying sample to the geometry. All measurements were performed in duplicate. Reported values were taken within the linear viscoelastic regime at 0.1% strain.

### 4.7 Microfluidics ‐ Droplet Linking in Flow

A microfluidic chip was designed to study linking of emulsion droplets in flow. The chip consisted of two distinct inlets for 1) the emulsion and 2) ultra‐pure water or 0.5 M NaCl solution. These two fluids intersected at the V‐junction of the chip, which allowed co‐flow towards bulb‐like chambers connected with narrow constrictions of 15 µm (Figure S12).

The master wafer was prepared using previously described UV‐lithography [65] and the protocol was adjusted to attain a channel height of 20 µm. The chip was made using polydimethylsiloxane (Sylgard 184 elastomer) and the curing agent was mixed in 10:1 weight ratio. The mixture was poured onto the master wafer, degassed using a vacuum desiccator and baked at 70°C for 12 h. The cured PDMS block was removed carefully and the inlet and exit holes were punched using a biopsy punch of 1 mm diameter (Darwin Microfluidics). The PDMS block was then plasma bonded on a glass slide (VWR) and incubated at 70°C for 2 h to ensure firm binding to glass slide.

To prevent emulsion droplets wetting the surface, the chip was passivated with a hydrophilic coating, by flowing a solution of 5 wt% polyvinyl alcohol (Sigma–Aldrich P8136) through the channels for approximately 2‐3 min and then removed using vacuum, ensuring no clogging in the channel [66]. The device was then baked at 120°C for 10 min on a hot‐plate and stored at room temperature in a dry and clean place. The device was rested for at least another 12 h, before performing the experiments.

To flow the respective fluids through the microfluidic channels of the device, PTFE tubing (LVF‐KTU‐15, Darwin microfluidic) was connected to a microfluidic reservoir XS (LVF‐KPT‐4XS, Darwin Microfluidics) and a pressure controller (OBK1‐MK3+, Elveflow). Channel 1 (0–2 bar) was used to flow particle‐stabilized emulsions and channel 2 was used to flow NaCl solutions with varying pH. Microscopic visualization of the fifth chamber in the chain was acquired using a Nikon‐Ti‐2 Eclipse inverted epifluorescence microscope equipped with Prime BSI Express camera and 10× air objective. The acquired images and time‐lapse were analyzed using Fiji 1.52 [67].

The emulsion used in the microfluidic chip was prepared following the procedure described before, except at a lower oil volume fraction ($\phi_{\mathrm{oil}}=$ 0.57), to prevent interdroplet linking caused by jamming prior to the experiment.

### 4.8 Interfacial Rheology

#### 4.8.1 Oil Stripping

Rapeseed oil was stripped from impurities using activated magnesium silicate powder (Florisil 100–200 mesh, Sigma–Aldrich) as these impurities might affect the interfacial measurements. The oil was stripped using a 3:1 (v:v) mixture of oil to Florisil and was stirred for 5 h with a head stirrer (Ultra‐Turrax, IKA, Germany) at 200 rpm. The oil mixture was then centrifuged at 10,000 g for 10 min after which the clear oil layer on top was collected and the remaining slurry was centrifuged again at 10,000 g for 30 min. The clear oil layer on top was collected and pooled with the previously collected oil. For the pellet, these steps were repeated once more, but stirring with Florisil was allowed to last overnight. The stripped oil was covered in aluminum foil and stored at −18°C to prevent oxidation.

#### 4.8.2 Pendant Drop Rheology

An automated drop tensiometer (Tracker, Teclis‐instruments, Tassin, France) was used to measure the interfacial tension of a droplet containing 0.01 wt% PPI in ultra‐pure water at pH 3 or 7. The drop tensiometer uses a drop shape algorithm to determine the interfacial tension between the two liquid phases, water and oil (stripped rapeseed oil), based on the Young‐Laplace equation:

(12) $$P_{L}=\sigma \left(\frac{1}{R_{1}}+\frac{1}{R_{2}}\right)$$

where $P_{L}$ is the Laplace pressure, σ the interfacial tension, and $R_{1}$ and $R_{2}$ the principal radii of curvature of the droplet.

The Teclis Windrop software was used to conduct the measurements, and the water bath was set to 20°C. A water droplet immersed in oil was used as opposed to an oil droplet immersed in water to ensure the protein dispersion did not obscure the drop curvature. Initially, the w/o interface of a water droplet at the end of an 18‐gauge needle in oil was left to equilibrate for 2 h at a constant interfacial area of 30 mm^2^. The interfacial tension of these droplets was compared to that of droplets containing only ultra‐pure water to calculate the reduction of interfacial tension by the pea proteins. Then, the drop volume and area were changed following sinusoidal oscillations of the interfacial area with increasing amplitudes (1%, 2%, 5%, 10%, 20%, 30%, 40%, 50%, and 60%). Under these conditions, the Worthington number (Wo), which describes the accuracy of interfacial tension measurements [68], was within an acceptable range (0.3–0.7) and viscous contributions of the fluids on the interface were negligible (Ca≪0.002) [69]. Five oscillations at 0.02 Hz were applied for each amplitude of which the middle three oscillations were used for analysis. Before moving to the next amplitude, the droplet was left to equilibrate for 10 min to negate any effects from previous oscillations. The WinDrop software was used to determine the dilatational storage and loss moduli ($E_{d}^{\prime }$ and $E_{d}^{\prime \prime }$) for the measured amplitudes. To gain intracycle information on interfacial dynamics, the normalized interfacial tension was plotted against the interfacial area change. All samples were measured in triplicate.

#### 4.8.3 Dynamic Thin Film Balance

A bike‐wheel chip with a custom‐designed microfluidic device based on the initial design of Cascao‐Perreira et al. [36] was used to study adhesive interactions between two opposing interfaces. Detailed information on the design and fabrication can be found elsewhere [37]. The pressure inside the chamber was controlled by a MK3+ OB1 piezoelectric pressure control system (Elveflow, France), with a pressure resolution of 1 Pa and a maximum pressure of 20 kPa. The pressure control was connected to the pressure chamber by rigid PTFE tubing with an inner diameter of 0.1 mm. The film was visualized using a Nikon Eclipse FN1 upright microscope equipped with a 10× long working distance objective (NA = 0.2) and was monitored by a Hamamatsu ORCA‐Flash4.0 V3 CMOS camera. A monochromatic wavelength of 622 nm was used for reflection. The sequence of images was saved (with a maximum of 10 ms temporal resolution) and then converted to thickness using Sheludko's equation [70]:

(13) $$h_{eq}\;=\frac{\lambda_{l}}{2\pi n_{f}}\left[m\pi \pm arcsin\sqrt{\frac{\Delta }{1+\frac{4Q(1-\Delta )}{{\left(1-Q\right)}^{2}}}}\right]$$

where $h_{eq}$ is the equivalent thickness, $\lambda_{l}$ is the wavelength of the monochromatic light used, $n_{f}$ and $n_{c}$ are the refractive indices of the thin film (water = 1.333) and outer phase (rapeseed oil = 1.470), respectively, and m is the order of interference. Δ and Q are equal to $\Delta =\frac{I_{f}-I_{min}}{I_{max}-I_{min}}$ and $Q={\left(\frac{n_{f}-n_{c}}{n_{f}+n_{c}}\right)}^{2}$ where $I_{f}$ is the intensity of the film and $I_{min}$ and $I_{max}$ the minimum and maximum intensities measured during the experiment. The microscope and the setup were placed on an active vibration isolation table (Vario Basic 60, Park Systems GmbH ‐ Accurion, Germany) to minimize the effect of external disturbances. Experiments were conducted at ambient room temperature (22 ± 0.1°C) and there was no observed effect of evaporation within the timescale of the experiments.

To study adhesion between interfaces we used dynamic measurements, in which a positive pressure step was followed by a negative one, as described in the protocol of Ref. [38]. This pressure profile was used to simulate the approach and separation of two droplets and measure adhesion between the droplets similar to Refs. [3] and [71]. This protocol is similar to that for droplet‐probe AFM measurements, except that here a constant pressure was applied instead of a constant velocity [72, 73]. Initially, an equilibrium pressure $P_{c,applied}$ was found by varying the pressure in steps of 1 Pa until the first interference rings appear (at film thickness of a few µm). This $P_{c,applied}$ was the sum of all contributions in the static thick film. Subsequently, the pressure inside the film was lowered using pressure drops, ΔP, of 100 Pa initiating film drainage. At a thickness of O(10^2^ nm), the hydrodynamic pressure builds up, causing radial expansion of the film. When the thin film reached a radius of ≈ 0.3 mm, the applied pressure was inverted, causing the inflow of liquid from the Plateau border to the film. The applied pressure profile is shown in Figure S13, and the detailed pressure balance with all relevant contributions are described in Ref. [39].

For the thin film measurements, PPI dispersions were prepared in ultra‐pure water at pH 3 and 7, and a protein concentration similar to the continuous phase of the emulsions (3.3 wt% protein). The dispersions were left to hydrate overnight while stirring (300 rpm) at room temperature. The next day, the dispersions were homogenized using high shear (Ultra‐Turrax, IKA, Germany) for 15 s at 8,000 rpm and filtered using a 0.2 µm Minisart NML Syringe Filter (Sartorius AG, Germany) just before measurement. The filtration ensured larger aggregates and particles were removed to prevent clogging of the channels.

### 4.9 3D Printing

To investigate the effects of droplet linking on mesoscopic material behavior, emulsions were printed using a FOOD Single 1.6 3D printer (FELIXprinters, IJsselstein, the Netherlands). To asses print fidelity and self‐standing properties, the emulsions were printed in the intricate shape of a Pokémon of approximately 4×4×4 cm with layers of 1 mm high using a nozzle of 2.5 mm in diameter. Prints were stored in the fridge and pictures were taken 1 hour after printing.

### 4.10 Statistical Analysis

Where applicable, the reported values are the average with standard deviation determined from triplicate or more measurements. For other experiments where averages could not be reported, like microscopic images and Lissajous‐Bowditch curves, data representative for the set of measured replicates was shown instead. Due to difficulty of the measurement, the evolution of standardized thin film radius versus standardized time was measured in duplicate and both replicates are reported.

## Funding

This study was funded by the Green Top Sectors TKI Call of 2022, with grant number: TKI OVK 22/293.

## Conflicts Of Interest

The authors declare no conflicts of interest.

## Supporting information


**Supporting File 1**: smll75117‐sup‐0001‐SuppMat.pdf.

**Supporting File 2**: smll75117‐sup‐0002‐VideoS1‐S6.zip

## Data Availability

The data that support the findings of this study are available from the corresponding author upon reasonable request.
