# Supplementary material for: Adhesive Protein Nanoparticles Control Emulsion Properties via Tunable Interdroplet Linking
Source: Small. 2026 Aug 12;22(61):e75117. doi: 10.1002/smll.75117 (PMC13725418; doi:10.1002/smll.75117)
Supplement: Supplementary file 1 [file SMLL-22-e75117-s002.pdf]

Supporting Information:
Adhesive Nanoparticles Control Emulsion Properties via Tunable Interdroplet Linking

G. D. Konings, Dr. K. A. Ganar, Dr. C. V. Nikiforidis
Biobased Chemistry and Technology, Wageningen University, Wageningen, Netherlands
Email Address: costas.nikiforidis@wur.nl
Dr. K. A. Ganar, Dr. E. Chatzigiannakis
Processing and Performance Group, Mechanical Engineering Department, Eindhoven University of Technology, Eindhoven, Netherlands
Dr. E. Chatzigiannakis
Institute of Complex Molecular Systems, Eindhoven University of Technology, Eindhoven, Netherlands
G. D. Konings, Prof. E. Scholten
Physics and Physical Chemistry of Foods, Wageningen University, Wageningen, Netherlands

S1. Emulsion morphology with (pH 3) and without nanoparticles (pH 7) and ionic strength.

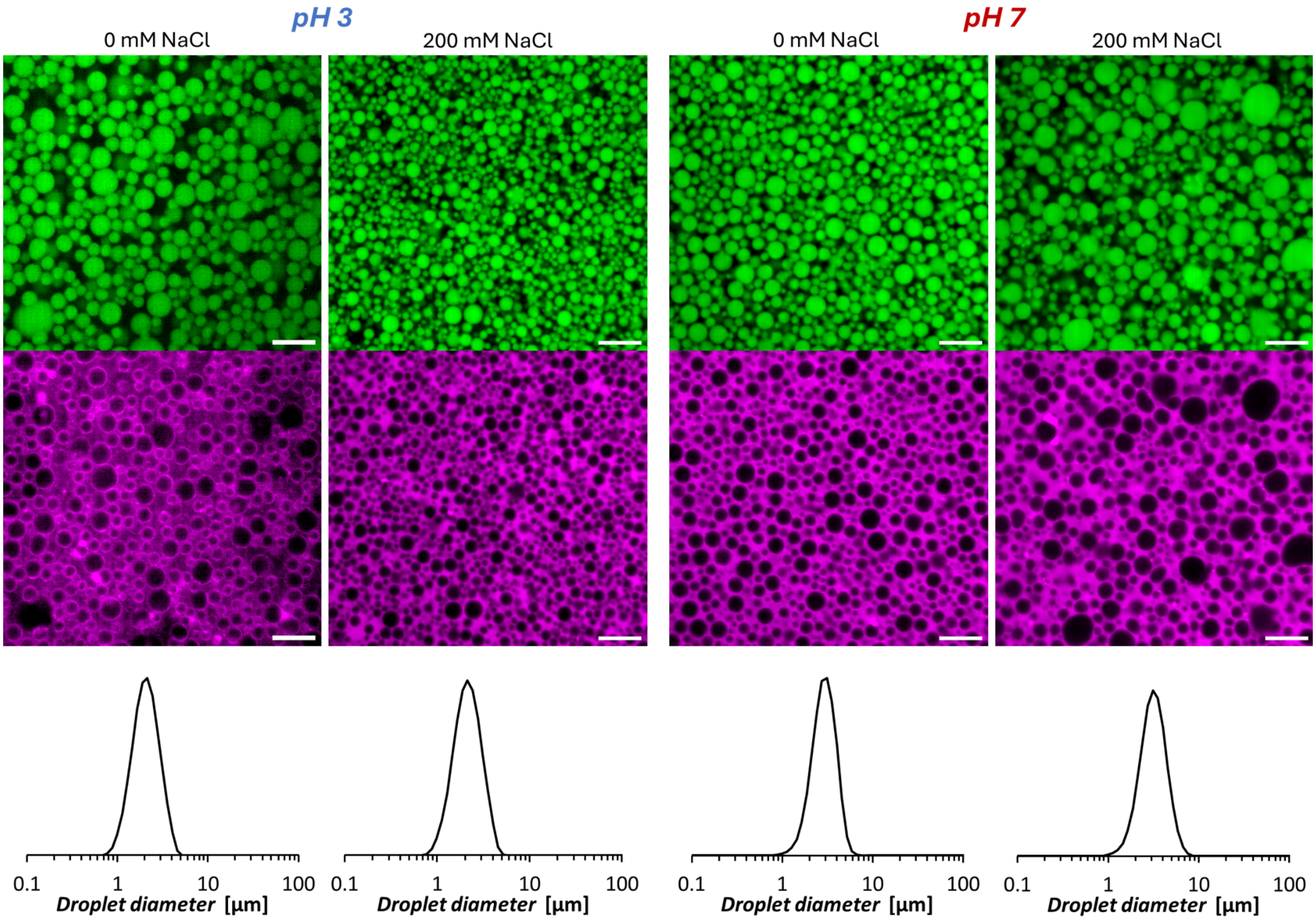

Figure S1. CLSM of emulsions with and without adhesive nanoparticles and in the absence of salt or with 200 mM NaCl in the continuous phase. Droplet size distribution is overlayed on top.

S2. Frequency sweep of jammed emulsions with (pH 3) and without nanoparticles (pH 7).

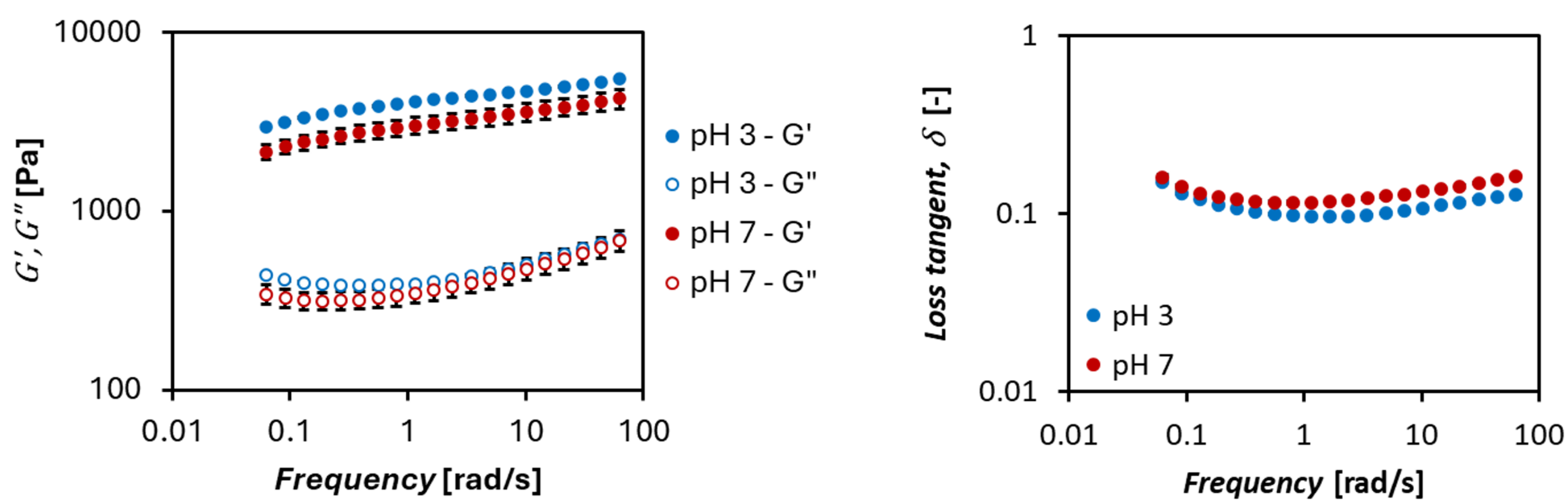

Figure S2. Frequency sweep of jammed emulsions with (pH 3) and without nanoparticles (pH 7). (**left**) Storage (G') and Loss (G") moduli across the probed frequency region (0.6283 to 62.83 rad/s) at a constant shear strain of 0.1%. (**right**) Loss tangent (δ) for the same emulsions under the same shear strain and probed frequency range. The presented data are the average of two replicates and the error bars show the standard deviation between the measured replicates. The emulsions measured consisted of 2 wt% pea protein and an oil volume fraction of 0.75.

S3. Relationship between emulsifying capacity, emulsion mechanical strength, and pH.

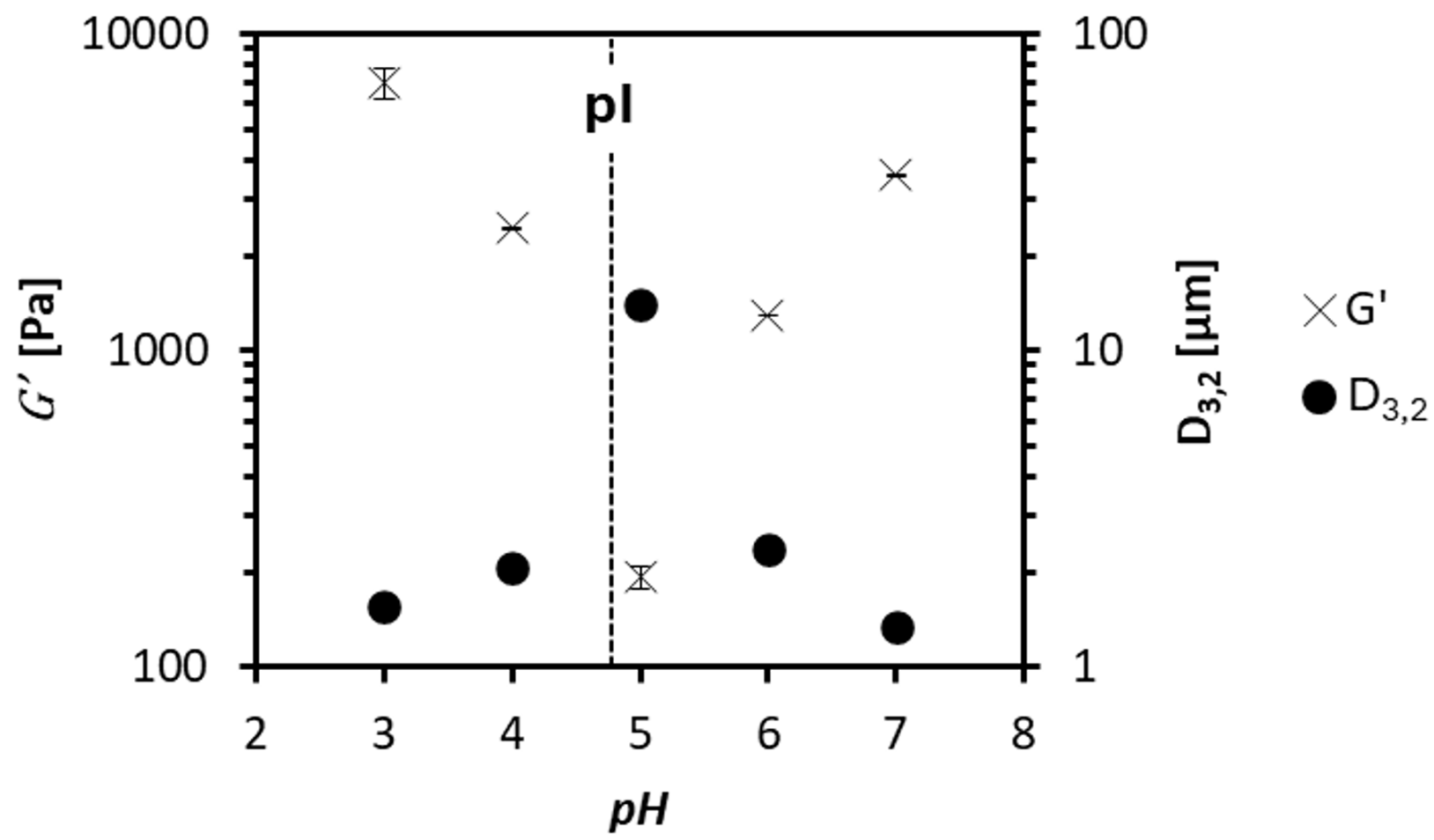

Figure S3. The emulsifying quality of a pea protein dispersion expressed as surface weighted average droplet size ($D_{3,2}$) and G' in the linear viscoelastic regime (0.1% strain) and a shear rate of 6.283 rad s^{-1}, as a function of pH. The isoelectric point (pI) is indicated by a dashed line. Emulsions were made with 2 wt% protein and had an oil volume fraction of 0.75.

S4. Emulsion droplet behavior with nanoparticles (pH 3) in flow at low ionic strength.

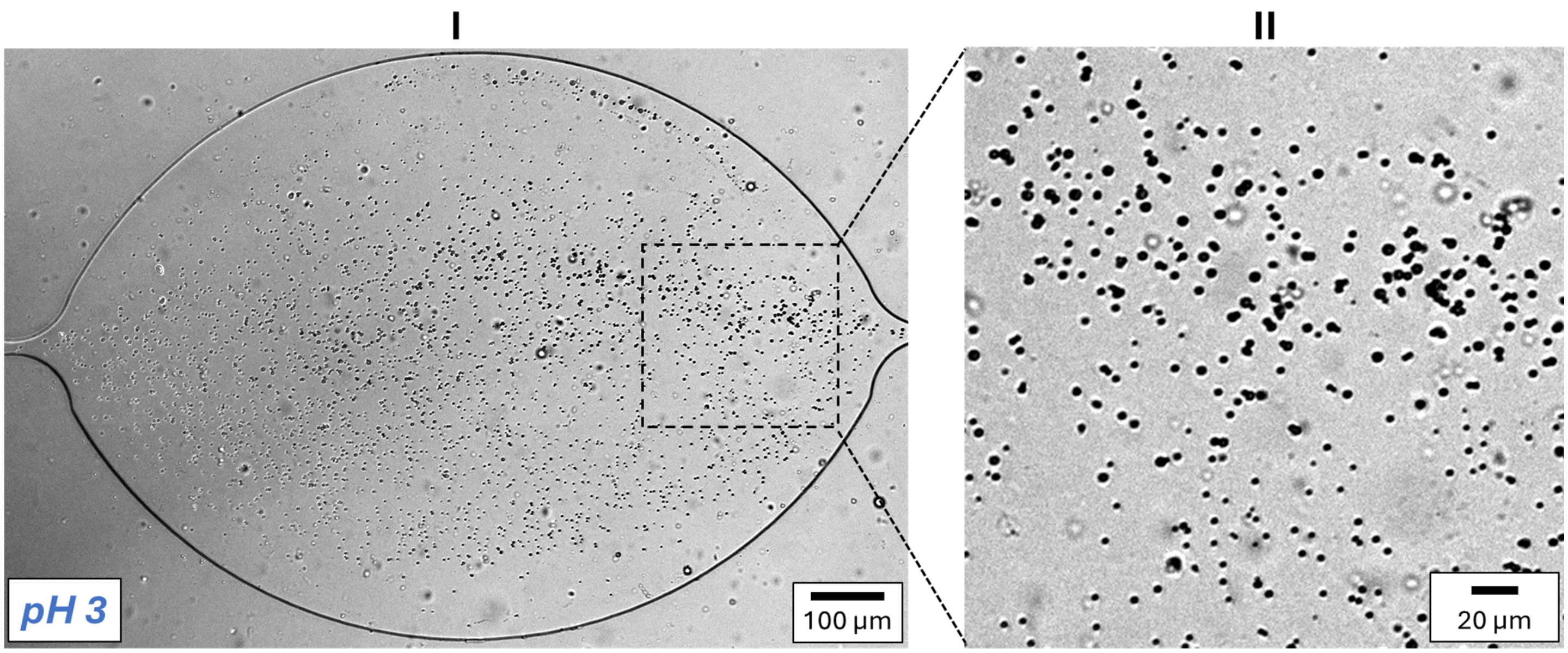

Figure S4. (**I**) Water and emulsion with nanoparticles (pH 3) co-flow through the chamber under an applied pressure of 25 mbar without aggregate formation. (**II**) Crop of individual droplets flowing through the channel.

S5. Emulsion droplet behavior without nanoparticles (pH 7) in flow at low ionic strength.

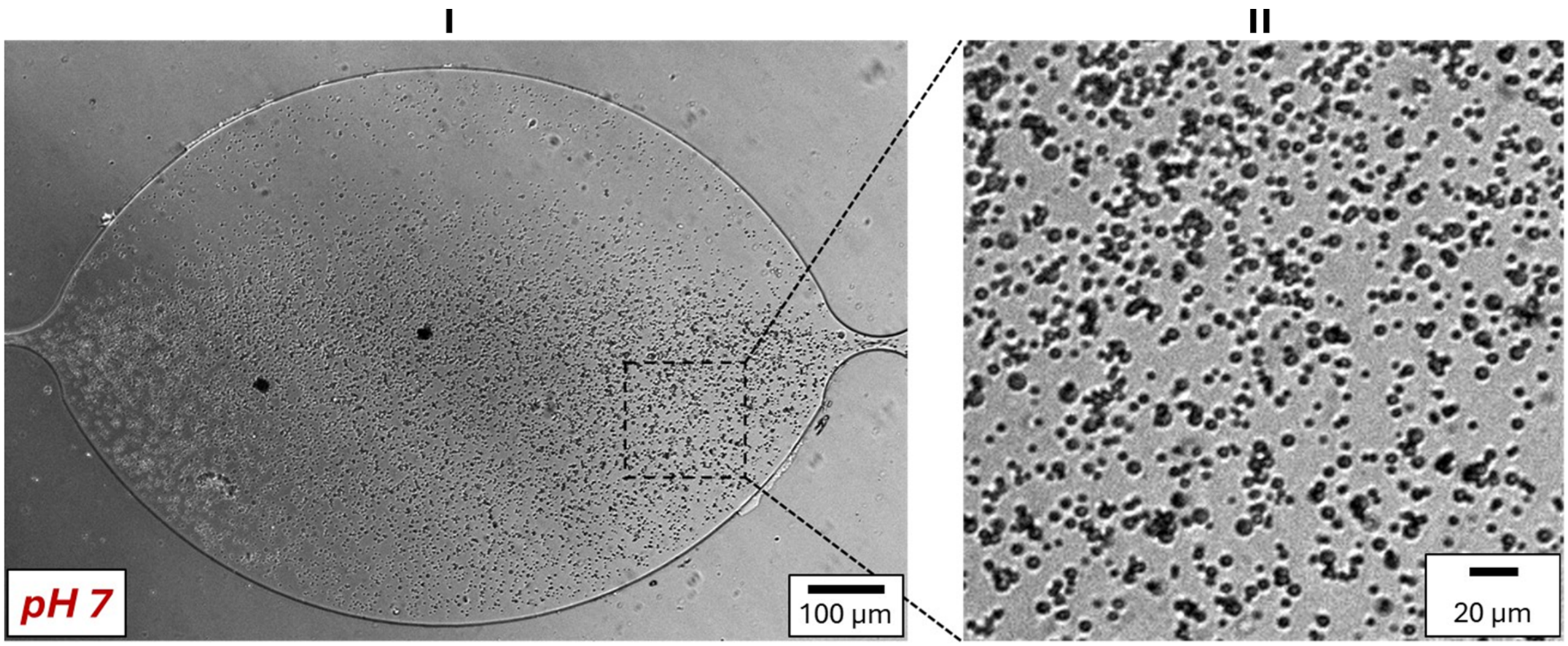

Figure S5. (**I**) Water and emulsion without particles (pH 7) co-flow through the chamber under an applied pressure of 25 mbar without aggregate formation. (**II**) Crop of individual droplets flowing through the channel.

S6. Surface potential trend of pea protein with pH and 200 mM NaCl for pH 3 and 7.

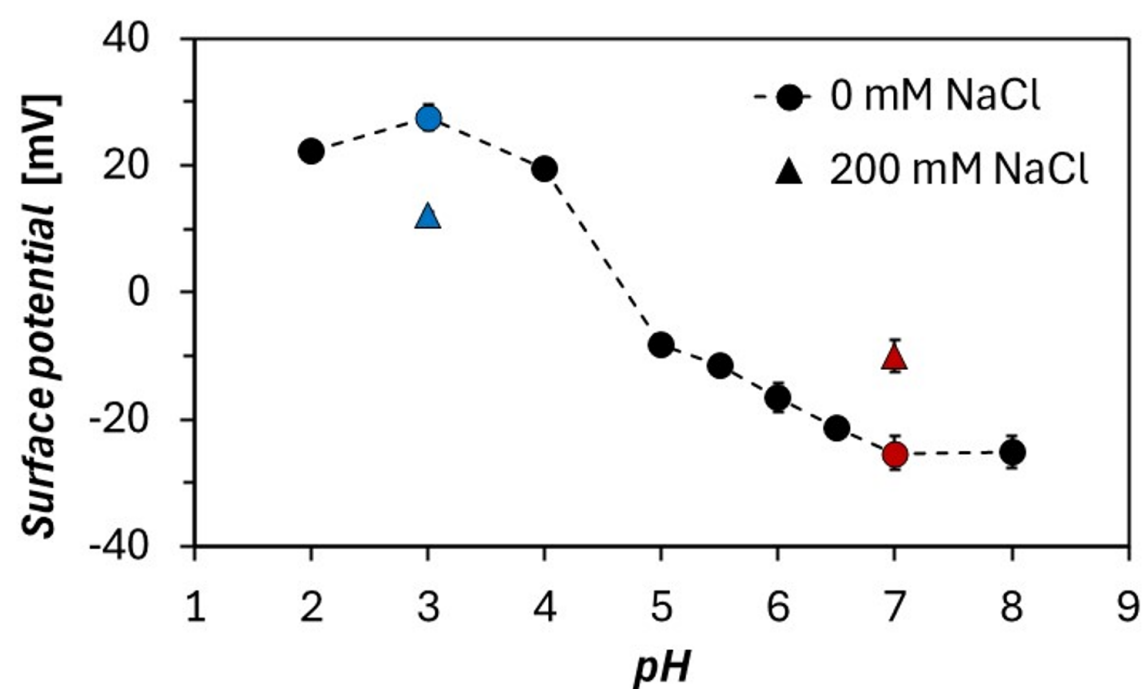

Figure S6. Surface potential of PPI dispersions over a range of pH and for pH 3 and 7 also with 200 mM NaCl.

S7. Droplet aggregation in flow for droplets without nanoparticles at high ionic strength.

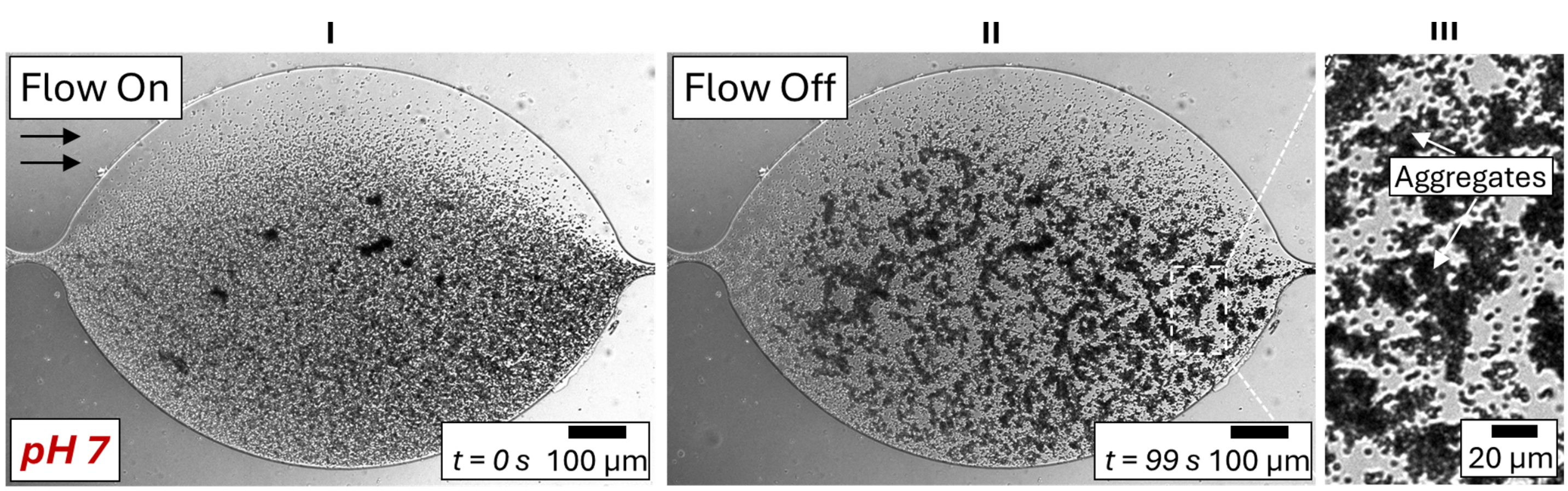

Figure S7. Real time aggregation of microemulsions without protein nanoparticles in presence of ≈0.2 M NaCl at pH 7. (**I**) NaCl solution and emulsions co-flow through the chamber at high flow rate (1000 mbar applied to both inlets) preventing aggregate formation. (**II**) Upon arresting flow (0 mbar), weak attractive forces between droplets exceed the shear forces induced by flow and emulsion droplets aggregate. (**III**) Zoomed-in view of the droplet aggregates.

S8. Micrograph of linked oil droplets and a schematic of the contact angle determination.

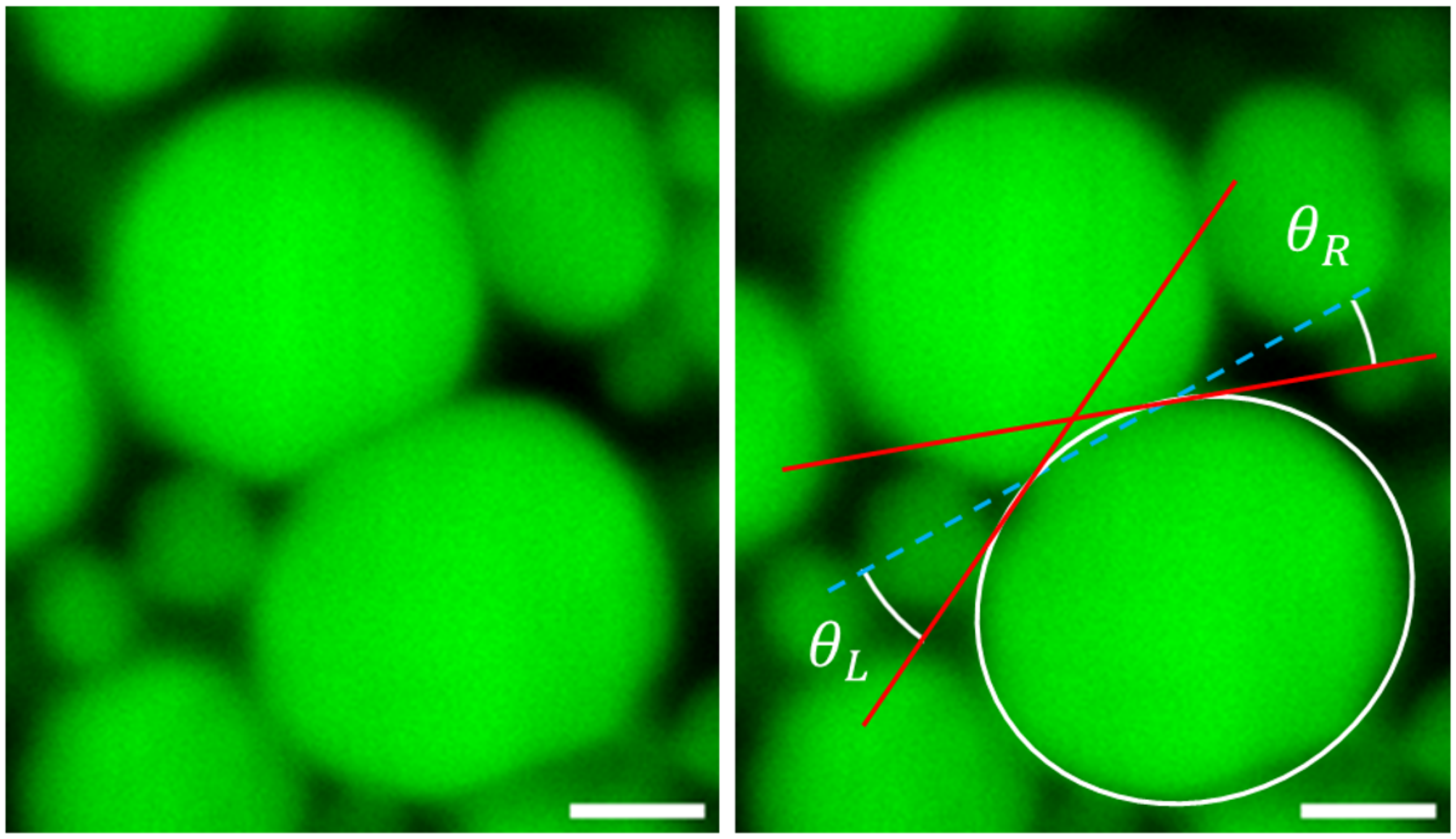

Figure S8. Two linked droplets (**left**), and a schematic showing how the contact angle was determined (**right**). The scale bar is 2 µm. A baseline was drawn along the flattened region where the two droplets made contact (blue dashed line), and an ellipse was fitted to the largest of the two droplets (white). From where the baseline and ellipse intersect, tangent lines were drawn (red). The angle between the tangent lines and the baseline was determined as the left (θ_L) and right (θ_R) contact angles, which when added together describe the adhesion angle ($2\theta_{adh}$).

S9. Protein particle size distribution at pH 3 and 7 for low and high ionic strength

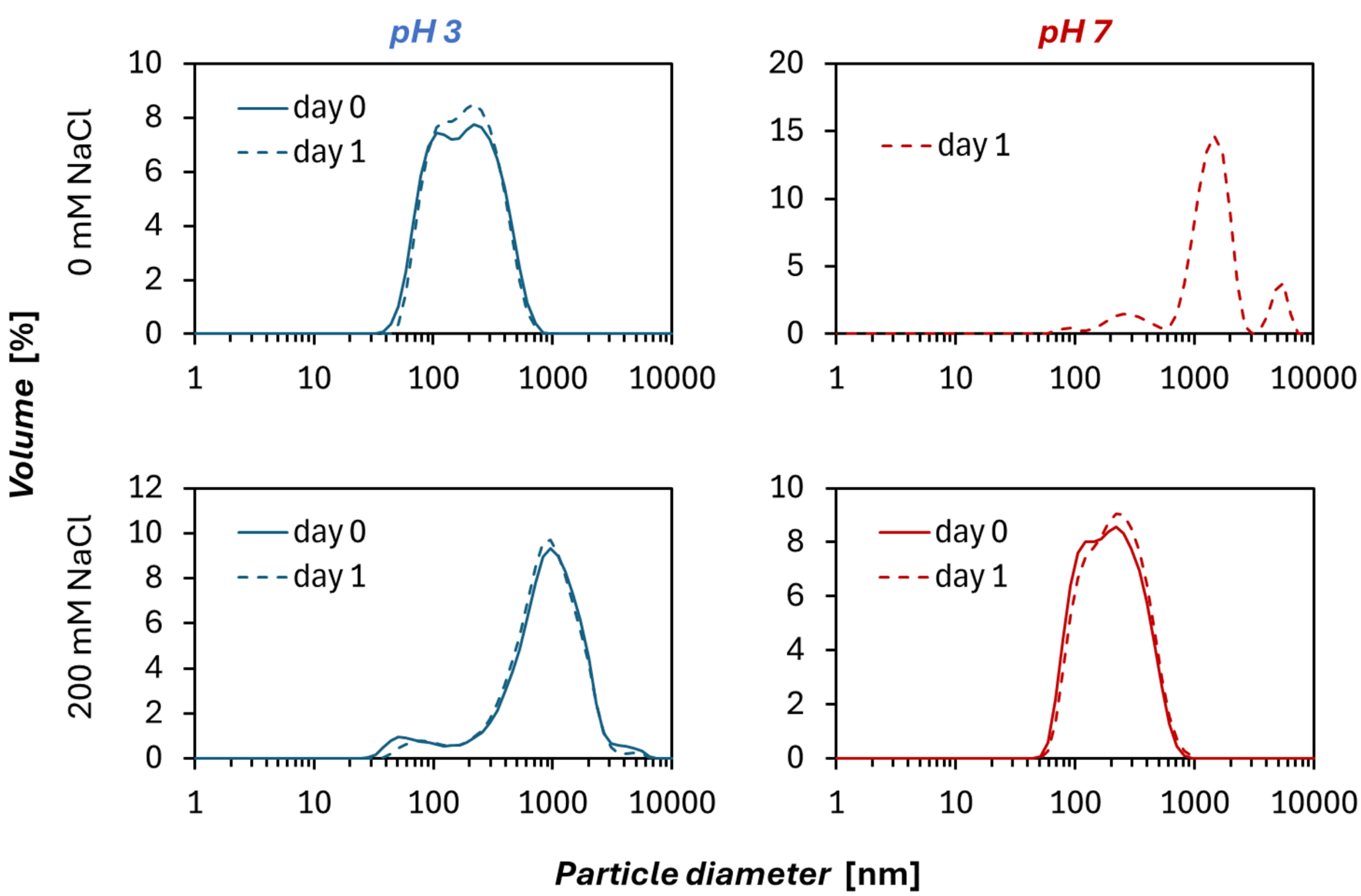

Figure S9. Particle size distribution of PPI dispersions for pH 3 and 7 with (200 mM NaCl) and without salt, both initially and after 1 day.

S10. Jamming transition of linked and non-linked emulsions.

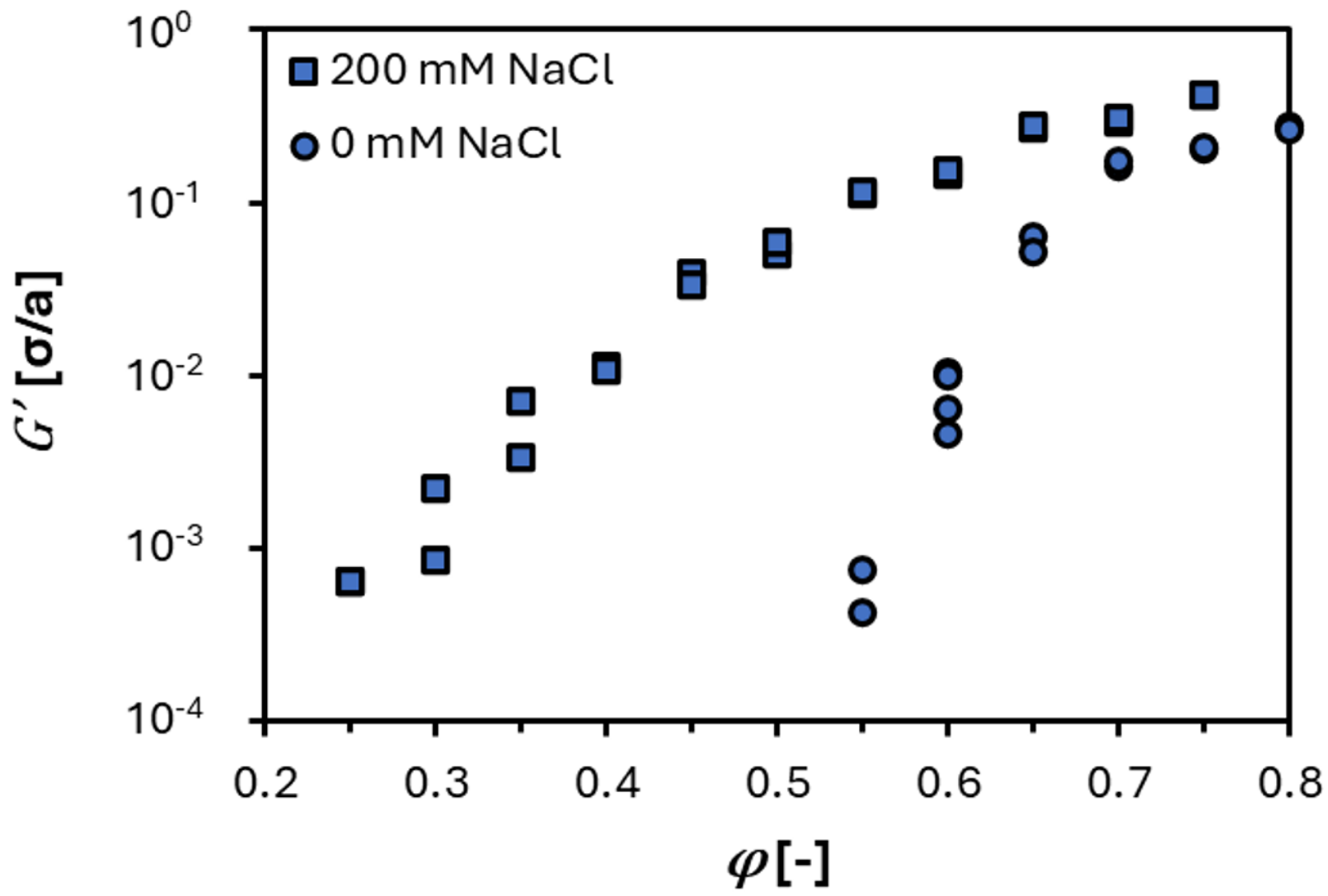

Figure S10. The jamming transition of linked (200 mM NaCl) and non-linked (0 mM NaCl) emulsions. The G' in the linear viscoelastic regime was scaled over units of Laplace pressure (surface tension, σ and, droplet radius, a) to better compare emulsions with different droplet size (minor changes between independently prepared samples) and surface tension (different ionic strength).

S11. Print stability and shape retention over time.

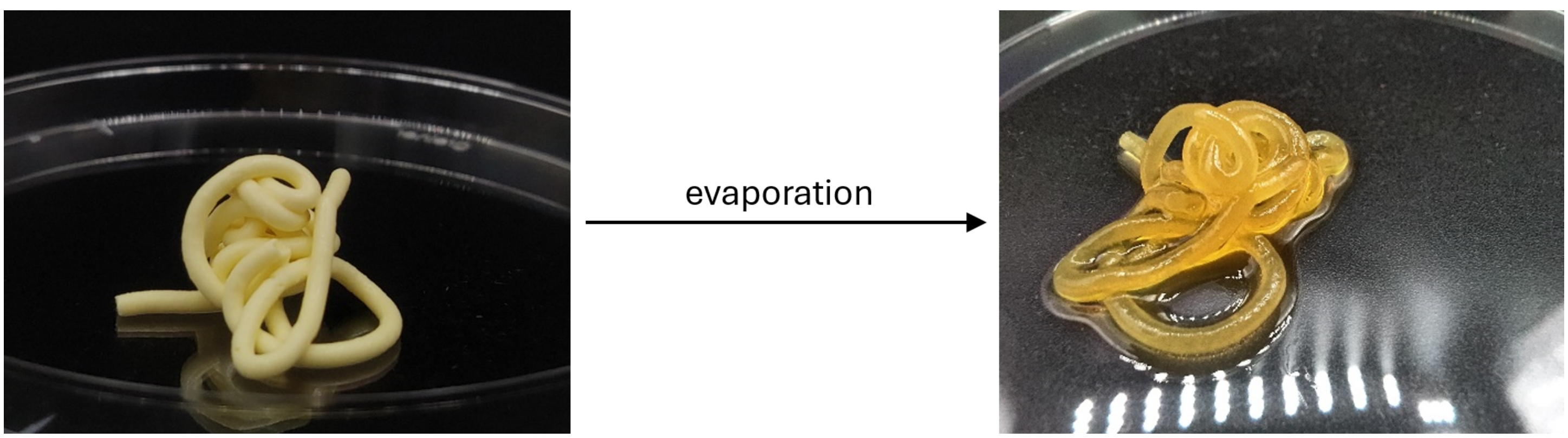

Figure S11. Emulsion at pH 3 and 200 mM NaCl and an oil volume fraction of 0.75 extruded by a syringe with a 2.5 mm nozzle diameter. (**left**) Picture taken immediately after extrusion, (**right**) a picture of the same extruded emulsion after evaporation in a fridge at 5°C for 8 days.

S12. Schematic of the microfluidic chip design for studying droplet linking in flow

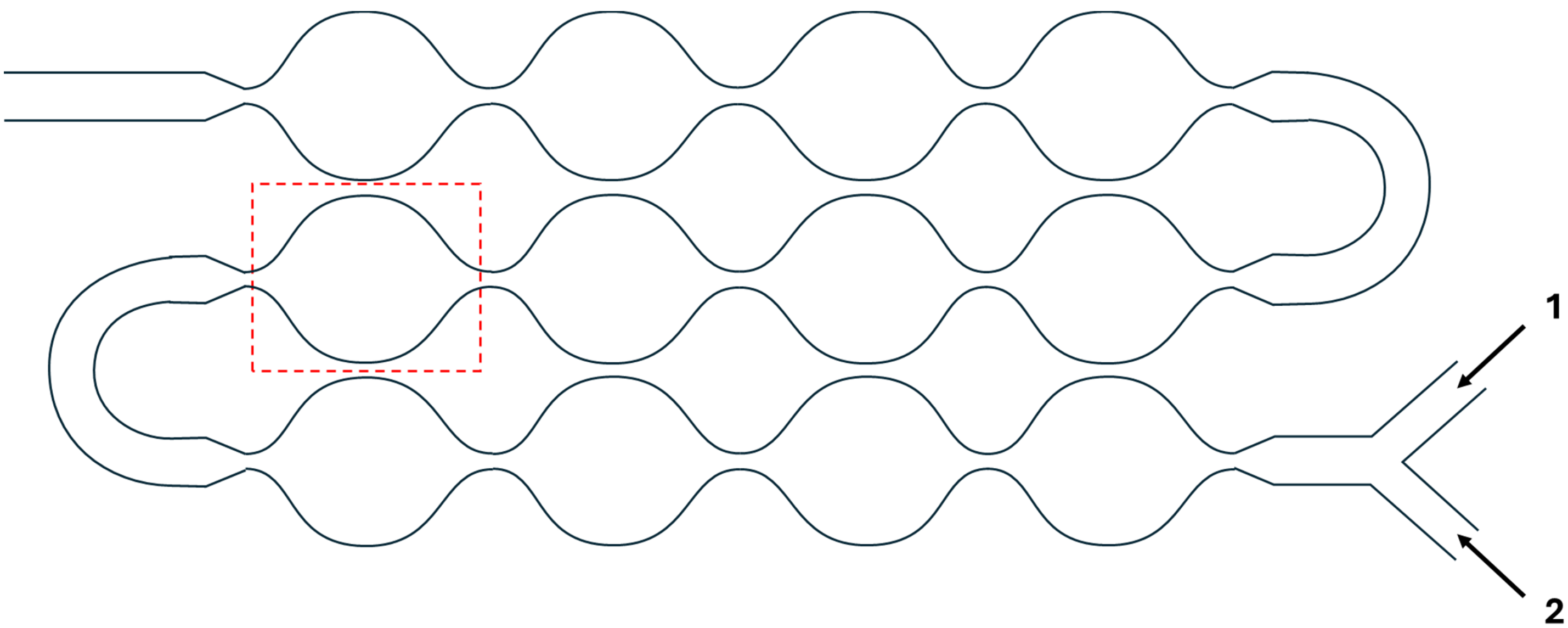

Figure S12. The layout of the channels and the captured region highlighted with a red dashed box. The emulsions came from inlet 1 and either the NaCl solution or ultra-pure water from inlet 2.

S13. The pressure profile applied in the thin film experiments

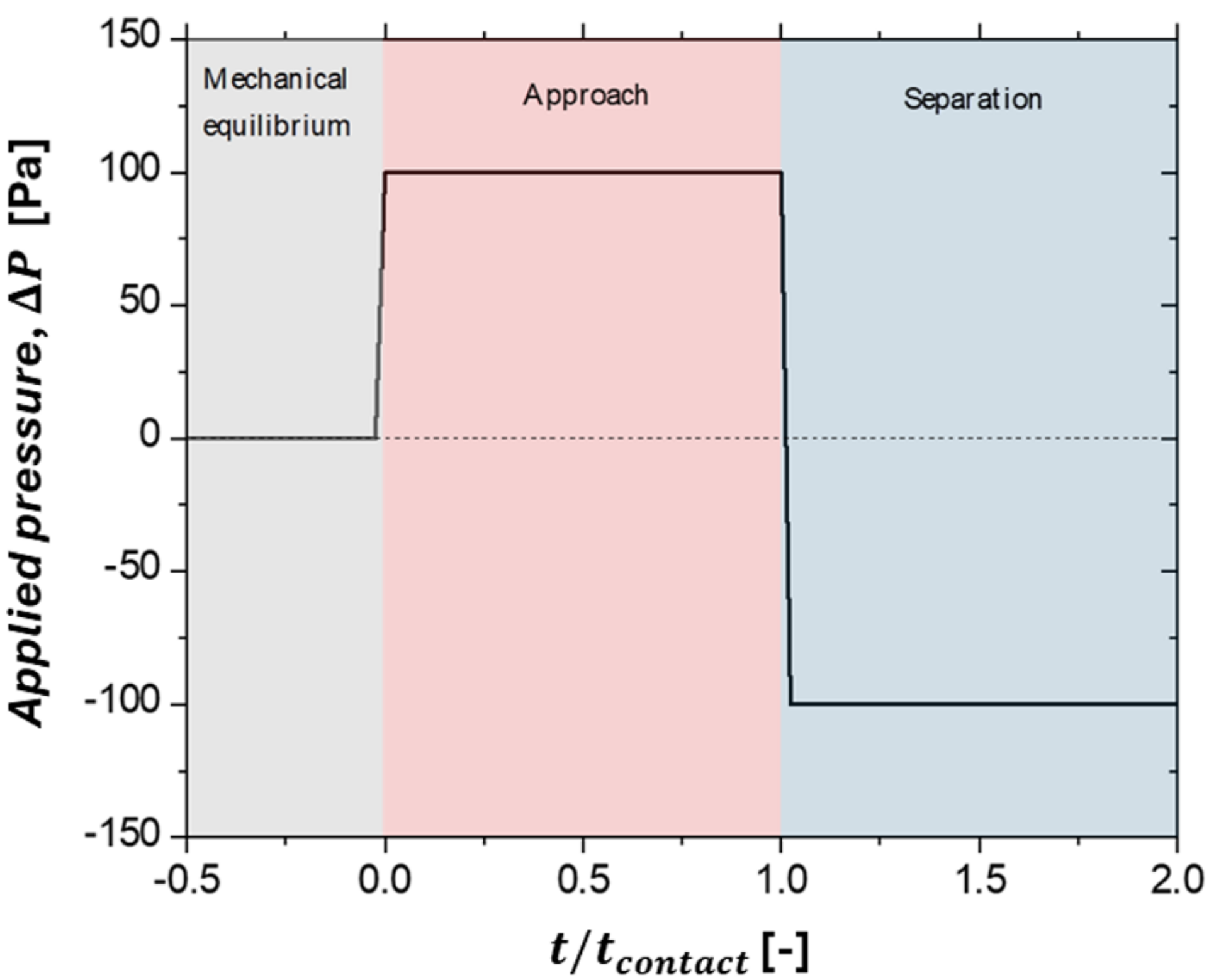

Figure S13. $P_{c,applied}$ for the dynamic measurements in the thin film balance normalized for contact time between the opposing interfaces.

Videos

Video 1. Shear-resistant interdroplet bridges prevent droplet aggregate dissociation despite constriction. Cropped view of droplet aggregates held together by adhesive protein nanoparticles flowing through a 10 μm narrow channel. Despite the aggregate being $> 5\times$ larger than the width of the constriction there was minimal aggregate dissociation, highlighting the shear-resistance of the droplet-bridges formed by pea protein nanoparticles. Video is available in a separate file.

Video 2. Dispersion of a droplet aggregate without adhesive nanoparticles by minor flow. Cropped view of a droplet aggregate without protein nanoparticles under minor flow. The aggregate was first lifted by the flow, and then quickly afterward, the aggregate disperses into individual droplets. The stark difference in behavior highlights the difference in adhesive forces constituted by the protein nanoparticles versus just the weak attractive van der Waals and hydrophobic forces holding the droplets together without the nanoparticles. Video is available in a separate file.

Video 3. Retarded separation rate of two 'oil droplets' with adhesive nanoparticles. Here, an aqueous film was formed between an outer phase of stripped rapeseed oil. This aqueous film contained the same mixture of pea protein and pea protein nanoparticles at pH 3 as the rest of the experiments, except larger nanoparticles (>0.2 μm) were filtered out. The video starts at a mechanical equilibrium close to formation of a thin film (~ 1 μm). Then, a positive pressure (ΔP) of 100 Pa was applied to the system, draining the aqueous film between the oil droplets. This positive pressure was applied until the thin film radius reached $\approx$0.3 mm. At this point, the applied pressure was inverted ($\Delta P = -100$ Pa), causing liquid to flow into the thin film from the Plateau border, separating the droplets. The rate of film drainage during approach, and film filling during separation were roughly equal. Without adhesion, one would expect a faster separation due to local changes in curvature of the droplets. This clearly shows the adhesive action of the nanoparticles present in the film. Video is available in a separate file.

Video 4. Fast separation rate of two 'oil droplets' without adhesive nanoparticles. Here, an aqueous film was formed between an outer phase of stripped rapeseed oil. This aqueous film contained the same pea protein dispersion at pH 7 as the rest of the experiments, except the dispersion was passed through a 0.2 μm filter (to match the preparation of the protein dispersion at pH 3). The video follows the same steps as for **Video 3**. In stark contrast to the thin film with nanoparticles, this film filled much faster ($\sim 4\times$) during separation, highlighting the lack of an adhesive force opposing separation. Video is available in a separate file.

Video 5. Pinned interfaces of two 'oil droplets' with adhesive nanoparticles and 0.2 M NaCl. Here, an aqueous film was formed between an outer phase of stripped rapeseed oil. This aqueous film contained a pea protein and pea protein nanoparticle dispersion at pH 3 which was prepared as before, except after overnight dispersion and high-shear homogenization, 2 M NaCl was added to a final concentration of 0.2 M NaCl. Afterward, the dispersion was filtered as before. The video follows the same steps as for **Video 3**. During the separation phase, signs of adhesion can be observed in the form of 'pinning', where the thin film periodically deviates from the typical circular shape. These deviations were caused by adhesive forces between the interfaces due to a reduced electrostatic repulsion. Video is available in a separate file.

Video 6. Thin film morphology of two 'oil droplets' without adhesive nanoparticles and 0.2 M NaCl during approach and separation. Here, an aqueous film was formed between an outer phase of stripped rapeseed oil. This aqueous film contained a pea protein dispersion at pH 7 which was prepared as before, except after overnight dispersion and high-shear homogenization, 2 M NaCl was added to a final concentration of 0.2 M NaCl. Afterward, the dispersion was filtered as before. The video follows the same steps as for **Video 3**. During the separation phase, signs of adhesion can be observed in the form of 'pinning', where the thin film periodically deviates from the typical circular shape. These de-

viations were caused by adhesive forces between the interfaces due to a reduced electrostatic repulsion. Video is available in a separate file.
